# Viral driven epigenetic events alter the expression of cancer-related genes in Epstein-Barr-virus naturally infected Burkitt lymphoma cell lines

**DOI:** 10.1038/s41598-017-05713-2

**Published:** 2017-07-19

**Authors:** Hector Hernandez-Vargas, Henri Gruffat, Marie Pierre Cros, Audrey Diederichs, Cécilia Sirand, Romina C. Vargas-Ayala, Antonin Jay, Geoffroy Durand, Florence Le Calvez-Kelm, Zdenko Herceg, Evelyne Manet, Christopher P. Wild, Massimo Tommasino, Rosita Accardi

**Affiliations:** 1 International Agency for Research on Cancer, World Health Organization, Lyon, 69372 France; 2 CIRI, (Oncogenic Herpesviruses Team), Lyon, France; 3 Inserm, U1111, Lyon, France; 4 Université Claude Bernard Lyon 1, CNRS, UMR5308 Lyon, France; 5 École Normale Supérieure de Lyon, Lyon, France; 6 Université Lyon, F-69007 Lyon, France

## Abstract

Epstein-Barr virus (EBV) was identified as the first human virus to be associated with a human malignancy, Burkitt’s lymphoma (BL), a pediatric cancer endemic in sub-Saharan Africa. The exact mechanism of how EBV contributes to the process of lymphomagenesis is not fully understood. Recent studies have highlighted a genetic difference between endemic (EBV+) and sporadic (EBV−) BL, with the endemic variant showing a lower somatic mutation load, which suggests the involvement of an alternative virally-driven process of transformation in the pathogenesis of endemic BL. We tested the hypothesis that a global change in DNA methylation may be induced by infection with EBV, possibly thereby accounting for the lower mutation load observed in endemic BL. Our comparative analysis of the methylation profiles of a panel of BL derived cell lines, naturally infected or not with EBV, revealed that the presence of the virus is associated with a specific pattern of DNA methylation resulting in altered expression of cellular genes with a known or potential role in lymphomagenesis. These included ID3, a gene often found to be mutated in sporadic BL. In summary this study provides evidence that EBV may contribute to the pathogenesis of BL through an epigenetic mechanism.

## Introduction

Epstein-Barr virus (EBV) is a double-stranded DNA herpesvirus, which infects 90% of the adult population worldwide with no adverse consequence for health in the majority of the cases^1^. However, more than 50 years ago, EBV particles were found in Burkitt’s lymphoma derived cultures^2^. This discovery resulted in the virus being recognized as the first human tumor virus. Since then, several epidemiological studies have shown that EBV is an etiological factor for endemic Burkitt’s lymphoma (BL) in Africa as well as of other human malignancies (such as nasopharyngeal carcinoma, gastric cancer, post-transplant lymphomas and some Hodgkin’s lymphomas)^1^. Nevertheless, the majority of individuals infected with EBV do not develop EBV-associated cancers, which suggests the involvement of additional genetic or environmental factors in the development of Burkitt’s lymphoma and other EBV-related cancers^3–7^. Well-recognized co-factors of EBV-induced malignancies include insect-borne parasitic infections like malaria, young age at first infection, immune suppression, and dietary factors. In the endemic variant of BL (eBL), EBV is found in each cancer cell, suggesting a direct role of EBV in the process of lymphomagenesis. However, other events, such as c-myc translocation are also required^8^. To date understanding of Burkitt’s lymphoma and the mechanistic role of EBV infection in the pathogenesis of this disease remain incomplete.

The development of omics technologies has enabled a fresh approach to the molecular characterization of EBV-induced malignancies and to further delineate the role of the virus in the process of transformation^9, 10^. The new technology has confirmed some of the previous findings, such as c-myc translocation being a hallmark of all Burkitt’s lymphomas, independent of the clinical variant or EBV-status. However, they also helped reveal novel BL-associated genetic alterations, such as protein-damaging sequence mutations affecting the ID3-TCF3 regulatory loop. It was shown that mutations that impair the inhibitory function of ID3 on proteins of the TCF family, leads to constitutive activation of B-cell signaling and to a cMYC-independent lymphoid proliferation^11, 12^. Moreover, ID3 knock-out mice showed a predisposition for lymphomagenesis in comparison to wild type mice^13^. Abate and colleagues recently showed that the ID3-TCF3 loop genes carry fewer mutations in the endemic (EBV+) than in the sporadic (EBV−) BL variant^14^. Overall, their data on RNA sequencing of eBL primary tumors revealed a lower rate of cellular mutations in genes previously found altered in sporadic BL (sBL) such as MYC and TP53. This highlights a potential role for non-genetic virally-driven events in the pathogenesis of EBV+ eBL.

Epigenetic modifications are important in cancer development and several lines of evidence suggest that certain oncogenic viruses have the ability to hijack enzymes that govern epigenetic modification, thereby altering the structure and function of the host genome^15–17^.

Recent studies have reported epigenetic changes occurring in B cells during the process of EBV-driven transformation. A profound epigenetic remodeling was also shown in EBV-driven epithelial cancers, such as Gastric Cancer (GC)^18^.

In the present study, we tested the hypothesis that the lower load of somatic mutation observed in eBL compared to the sBL variant can be explained by abnormal DNA methylation induced by infection with EBV. Our results show that EBV modifies the epigenetic profile of the B cell genome and as a consequence alters the expression of genes with a known or potential role in lymphomagenesis, supporting a direct role of the virus in the pathogenesis of eBL.

## Results

### The methylome landscape of EBV+ Burkitt’s lymphomas derived cell lines

**Figure 1:**
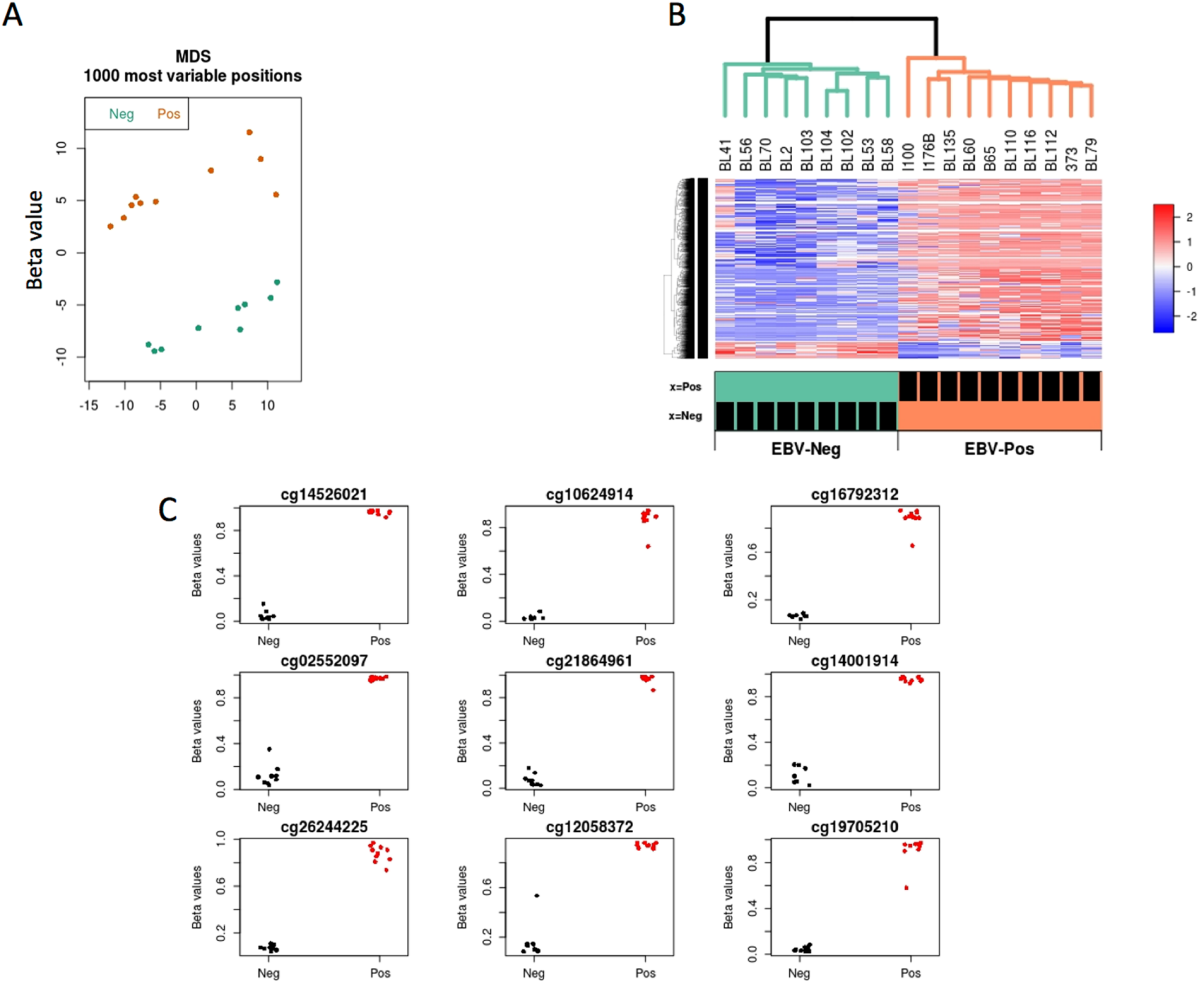
EBV-dependent methylation at the single CpG site level. BL samples were processed for genome-wide methylation analyses using HM450 bead arrays, as described in Methods. (**A**) Multi-dimensional scaling (MDS) plot showing two main groups of samples, generally matching EBV status. (**B**) Heat map of differentially methylated positions (DMPs) between the two EBV categories. (**C**) Example stripchart plots of the top most significant differentially methylated positions shown.

To identify a potential impact of EBV on DNA methylation patterns in Burkitt Lymphoma (BL), we first profiled the DNA methylome of 10 EBV (+) and 9 EBV (−) BL-derived human cell lines. The EBV (−) BL cell lines derived from BL samples from individuals of Caucasian origin and display a very low number of EBV copies (from 0.02 to 0 copies per cell) when analysed by Taqman PCR, while the EBV (+) BL were almost all derived from BL samples from individuals of African origin and displayed at least 1 copy of EBV genome per cell (Supplementary Table 1). DNA was bisulfite converted and interrogated for DNA methylation using Illumina HM450 bead arrays (as described in Methods). Data quality was ensured by verifying internal standards, filtering out low quality or cross-reactive probes, and using multi-dimensional scaling to rule out batch effects. Interestingly, our initial multidimensional scaling (MDS) plot revealed that EBV status was the single most important variable defining variation in DNA methylation data (Fig. 1A). This was further supported by the unsupervised hierarchical clustering that was able to discriminate the samples into two discrete groups, perfectly segregated by EBV status (Fig. 1B).

After normalization, linear regression was used to define differential methylation at single-locus and regional levels. To further increase the stringency of this analysis, we defined a differentially methylated position (DMP) as a significant change in mean methylation of at least 40% between the two conditions (FDR adjusted P value < 0.05). 4712 DMPs were found using these criteria, with 453 hypomethylated and 4259 hypermethylated in EBV+ samples (Fig. 1C and Table 1).

**Table 1:** Differentially methylated positions (DMPs). Top 100 most significant DMPs are shown (FDR < 0.05, delta-beta >40%).

Target ID	adj.P.Val	distance	nearest Gene Symbol	nearest TSS
cg10624914	0	0	ADPRHL1	ADPRHL1
cg26517376	1E-07	0	FAM53B	FAM53B
cg22478679	2E-07	0	ADPRHL1	ADPRHL1
cg19705210	3E-07	8657	CCDC141	CCDC141
cg00767058	8E-07	0	ADPRHL1	ADPRHL1
cg00318643	8E-07	54721	ACSF3	ACSF3
cg13039251	9E-07	0	PDZD2	PDZD2
cg21864961	1.2E-06	18820	FBXL14	WNT5B
cg09982224	1.9E-06	74	ALDH3B1	ALDH3B1
cg19364276	2.2E-06	26371	LONRF2	LONRF2
cg03502979	3.4E-06	0	ADPRHL1	ADPRHL1
cg26244225	4.9E-06	0	APOLD1	APOLD1
cg07458509	7.7E-06	0	CD320	CD320
cg22697034	9.1E-06	33017	ABR	ABR
cg26112170	1.19E-05	0	ADPRHL1	ADPRHL1
cg03127370	2.02E-05	1306	NTN3	NTN3
cg16628205	2.02E-05	0	TFR2	TFR2
cg05669853	2.52E-05	0	BEND3	BEND3
cg00963675	2.81E-05	0	EGR2	EGR2
cg04398180	3.86E-05	0	ADPRHL1	ADPRHL1
cg25325005	3.92E-05	0	PLEC	PLEC
cg08625693	0.00004	0	DLG3	DLG3
cg08315421	0.00004	0	S100Z	S100Z
cg03078488	4.08E-05	0	IGF2BP3	IGF2BP3
cg25202367	4.96E-05	0	CDK19	CDK19
cg04848686	4.96E-05	0	SNAI3	SNAI3
cg26600461	4.96E-05	58252	CBFA2T3	CBFA2T3
cg22449745	4.96E-05	0	C1orf109	C1orf109
cg03895159	5.22E-05	0	KLHL24	KLHL24
cg25594106	5.22E-05	6659	SNX6	EAPP
cg22101098	6.06E-05	0	SLC17A1	SLC17A1
cg24278165	6.06E-05	0	LOC389641	LOC389641
cg02789394	6.06E-05	0	FYN	FYN
cg12057368	6.33E-05	0	RCC1	RCC1
cg10016175	6.52E-05	0	SNAI3	SNAI3
cg18198550	6.92E-05	935	SRPK1	SRPK1
cg03178454	6.99E-05	0	KCNH2	KCNH2
cg06194602	7.39E-05	857	GH1	GH1
cg02190383	7.39E-05	0	BEST1	BEST1
cg26354221	7.66E-05	0	SPECC1L	ADORA2A
cg12481266	8.11E-05	0	MFSD4A	MFSD4A
cg04618812	8.27E-05	0	ACADS	ACADS
cg23088510	8.39E-05	0	FAM53B	FAM53B
cg11851129	8.39E-05	0	SHMT2	SHMT2
cg23035330	9.51E-05	0	SPTBN1	SPTBN1
cg09560953	0.000101	572	UBE2E1	UBE2E1
cg16838967	0.000101	0	PLD4	PLD4
cg15510325	0.000101	0	KCNH2	KCNH2
cg05197508	0.000103	67629	STARD13	STARD13
cg12196685	0.000103	0	AMZ2P1	AMZ2P1
cg04313941	0.000103	12069	LOC645752	LOC645752
cg03279633	0.000103	2000	ZNF827	ZNF827
cg00602811	0.000103	98	ZEB2-AS1	ZEB2
cg10131879	0.000103	0	GLCCI1	GLCCI1
cg06539276	0.000103	2117	ZNF655	ZNF655
cg23303108	0.000103	0	LOC389641	LOC389641
cg19924334	0.000105	0	HELZ2	HELZ2
cg09087087	0.000105	18884	FBXL14	WNT5B
cg14033585	0.000105	11333	UBE2Q2	UBE2Q2
cg15604357	0.000113	0	VANGL2	VANGL2
cg22764289	0.000113	0	SYBU	SYBU
cg05993778	0.000118	0	TOMM5	TOMM5
cg19377421	0.000118	0	ISG20L2	ISG20L2
cg18133477	0.000121	0	TP53INP2	TP53INP2
cg13549345	0.000121	0	NACC2	UBAC1
cg08410533	0.000125	0	DIP2C	DIP2C
cg01209199	0.000125	0	CRIP3	CRIP3
cg06256007	0.000125	101588	SOX6	SOX6
cg04955573	0.000125	0	GFI1	GFI1
cg14784253	0.000126	0	NOD1	NOD1
cg10039500	0.000129	0	ADPRHL1	ADPRHL1
cg18401111	0.000129	2270	OTUD7A	KLF13
cg13959241	0.000129	0	NACC2	UBAC1
cg05163330	0.000131	0	ADPRHL1	ADPRHL1
cg09056876	0.00014	380664	ARID1B	ARID1B
cg04880804	0.00014	0	PRSS27	PRSS27
cg22586884	0.00014	1233	PLPP1	PLPP1
cg00698688	0.00014	0	SULT2B1	SULT2B1
cg26002091	0.000144	0	DNMBP	DNMBP
cg25064551	0.000167	4082	DCAF4L1	DCAF4L1
cg00571819	0.000167	0	FUBP3	FUBP3
cg25592206	0.000167	0	CDKN2C	CDKN2C
cg14578677	0.000167	1331	TLR6	TLR6
cg10531355	0.000169	0	SERINC5	SERINC5
cg12362077	0.000169	619	STK35	STK35
cg06112560	0.000181	0	PLK1	PLK1
cg17478282	0.000181	139	TTC24	TTC24
cg10753398	0.000203	772	ZFP36	ZFP36
cg26246572	0.00021	41272	FNDC1	FNDC1
cg12067421	0.00021	4544	CHIT1	CHIT1
cg14373988	0.00022	1322	PEX10	PEX10
cg23660197	0.00022	0	MICB	MICB
cg12769519	0.00022	8211	LOC100996291	TMEM235
cg08661112	0.00022	347	PANK1	PANK1
cg04611493	0.00022	26392	LONRF2	LONRF2
cg09779405	0.00022	110338	TMCO5A	TMCO5A
cg24032890	0.000242	0	GNPTAB	SYCP3
cg12058372	0.000257	0	B4GALT5	PTGIS
cg12710480	0.000265	0	NECAB3	ACTL10
cg20964216	0.000265	33195	ABR	ABR

**Figure 2:**
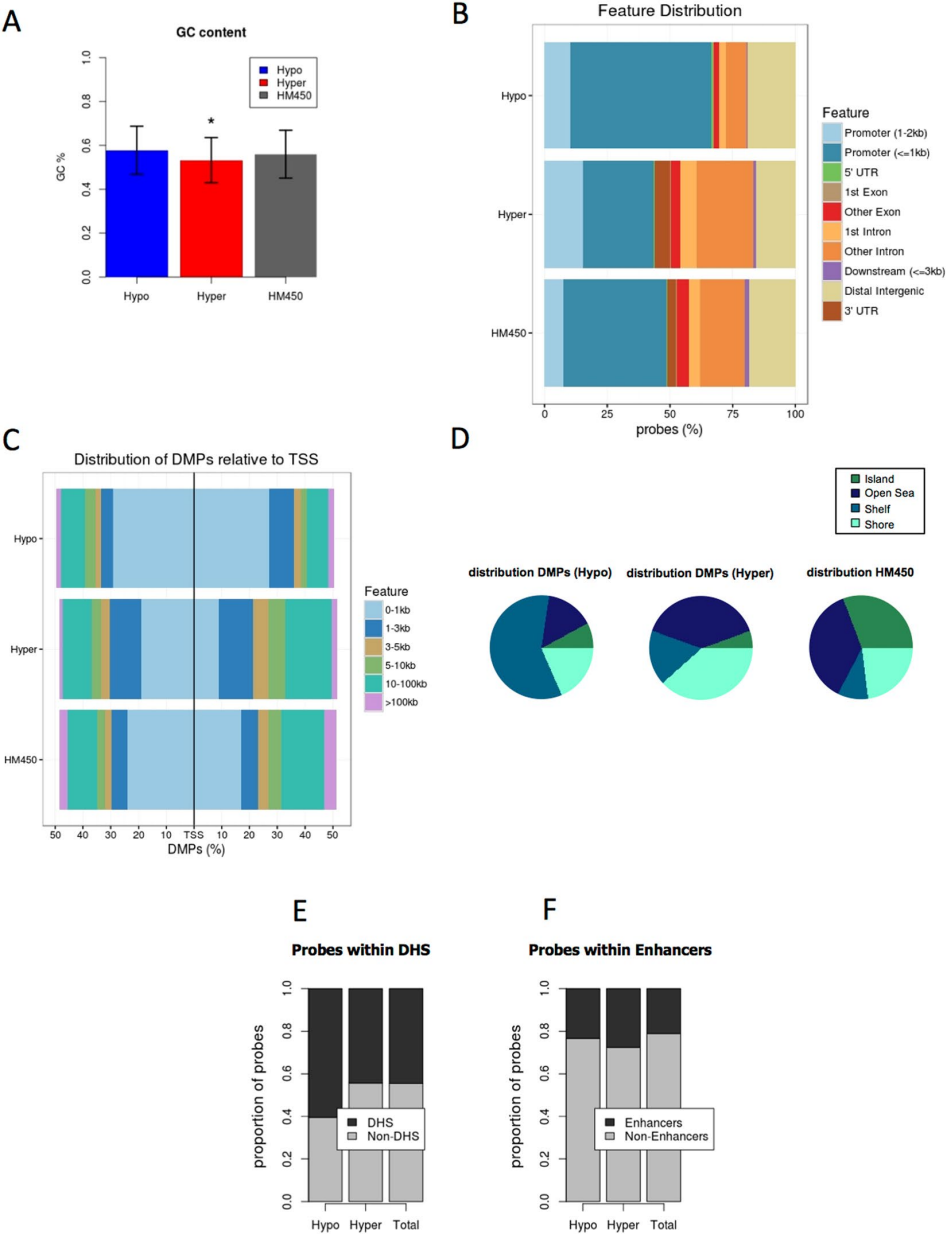
Genomic distribution of differentially methylated positions (DMPs). Differentially methylated positions (DMPs) were obtained after comparing DNA methylation profiles of EBV+ and EBV− Burkitt lymphoma-derived cell lines. DMPs were defined as hypo or hypermethylated in EBV+, relative to EBV− cells. (**A**) The percentage of GC content was calculated for each set of probes (i.e. hypomethylated, hypermethylated, and total HM450). (**B**) hypo and hypermethylated DMPs were mapped to different gene locations and their proportions represented with different colors. The total content of the Illumina beadchip (HM450) is shown for comparison. (**C**) Proportion of hypo and hypermethylated DMPs and total HM450 probes mapping to different distances from their closest transcription start site (TSS). (**D**) Location of hypo and hypermethylated DMPs and total HM450 probes relative to CpG islands. Colors represent the proportion of probes mapping to islands, shores (2 kilobases up and downstream from islands), shelves (2 kilobases up and downstream from shores), and open sea (more than 4 kilobases away from any island). (**E**) Proportion of hypo and hypermethylated DMPs and total HM450 probes mapping to DNAse hypersensitive sites (DHS). (**F**) Proportion of hypo and hypermethylated DMPs and total HM450 probes mapping to enhancer regions. (*) P value < 0.05.

Hypomethylated and hypermethylated DMPs displayed a distinct genomic distribution. The percentage of guanine-cytosine (GC) content was calculated for each set of probes (i.e. hypomethylated, hypermethylated, and total HM450 probe set). On average, hypermethylated sites were low in GC content, while hypomethylated sites displayed a similar GC content as the whole probe set represented in the HM450 arrays (Fig. 2A). Hypo and hypermethylated DMPs were mapped to different gene locations (i.e. promoter, 5′UTR, intron, exon, 3′UTR, downstream or intergenic). A significant hypomethylation was common in promoter regions (Fig. 2B), 0–1 kb from transcriptional start sites (TSS) (Fig. 2C). Location of hypo and hypermethylated DMPs and total HM450 probes relative to CpG islands showed that hypomethylated DMPs were enriched in CpG islands (Fig. 2D). Hypomethylated regions also showed an increase in DNase hypersensitive site (DHS) (Fig. 2E). On the contrary, hypermethylated sites were significantly more distant from TSS (Fig. 2B and C), and enriched in CpG island shelves (4 kilobases up and downstream from islands), with a trend to be localized in enhancer regions (Fig. 2D and F). Of note, many of the identified DMPs corresponded to the same gene symbols (Table 1), indicating that changes associated with EBV infection were spanning larger genomic regions.

**Figure 3:**
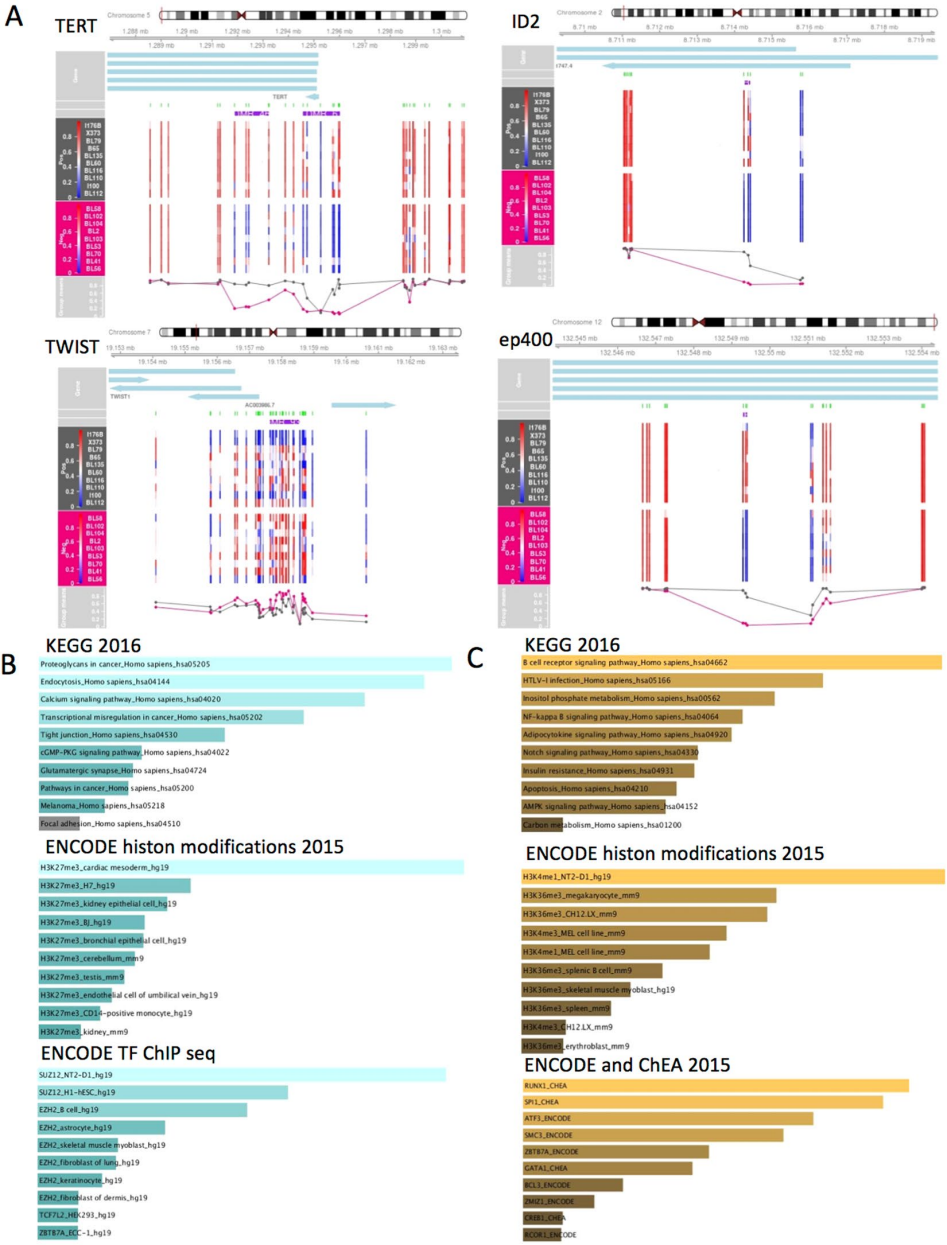
EBV-dependent methylation at the regional level and analysis of pathways targeted by EBV-dependent hyper or hypo methylation. (**A**) DMR plot corresponding to 4 of the EBV-associated DMRs. (**B**,**C**) Pathway analyses of EBV-associated DMRs discriminated by hypo (**B**) or hypermethylation (**C**). Enrichr web tool was used with the genomic locations of significant DMRs. Enrichment results are shown for the indicated databases (KEGG, BioCarta and ENCODE TF ChIP-seq).

To further investigate regional differences in methylation we performed region-level analysis in the same dataset. With a DMR (differentially methylated region) definition of at least 2 differentially methylated sites and a gap of less than 1000 bp, we identified 966 DMRs (Table 2). DMRs had an average of 6 CpG sites and an average size of 622 bp. Of these, 188 were hypomethylated DMRs and 778 were hypermethylated. Of note, the differentially methylated regions included genes with a well-known role in lymphomagenesis, such as ID2, or Twist, that has been shown to interact with the ID transcription factors^19^, as well as other cancer related genes, such as the Telomerase Reverse Transcriptase TERT (Fig. 3A).

**Table 2:** Differentially methylated regions (DMRs).

nearest Gene Symbol	distance	no. cpgs	minfdr	Stouffer	maxbetafc	meanbetafc
WDR46	0	90	0	0	0.789385	0.293411
ZBED9	0	59	0	0.066475	−0.51006	−0.23461
TBX3	12178	53	0	0.11526	−0.41401	−0.23084
EDNRB	0	39	0	0.123934	−0.54122	−0.27327
HLA-J	0	38	0	0.001062	−0.4929	−0.26044
ZBED9	47430	36	0	0.013715	−0.5175	−0.29018
CCNA1	0	33	0	2.1E-06	−0.58132	−0.33155
HLA-F-AS1	0	33	0	0.141073	−0.44396	−0.21893
UBD	1585	32	0	0.354622	−0.48394	−0.27808
SLC44A4	0	27	0	0.010108	0.574688	0.219413
GATA3-AS1	0	27	0	0.416264	−0.44296	−0.2351
TIMM17B	0	23	0	0.004317	0.387342	0.214287
IKBKG	0	21	0	8.27E-05	0.394816	0.232118
NEUROG1	0	21	0	0.145058	−0.52722	−0.25224
VARS	0	21	0	0.584609	0.446223	0.155537
HOXB4	0	20	0	0.000691	−0.46372	−0.2623
MSX1	693	20	0	0.001906	−0.46058	−0.26393
COL11A2	0	20	0	0.40899	0.694227	0.187249
PEX10	403	19	0	1E-07	0.693569	0.326587
TNNI2	0	19	0	0.000471	0.728032	0.174256
HTATSF1	0	19	0	0.003658	0.310737	0.167291
HCP5	0	19	0	0.178165	−0.29116	−0.0966
GATA5	290	18	0	0.01699	−0.53699	−0.26472
KIFC1	120	18	0	0.867494	0.687666	0.101494
PEX11A	0	17	0	0	−0.42046	−0.31884
MSC	0	17	0	0.000116	−0.51226	−0.37388
TMEM254	0	17	0	0.001087	−0.45833	−0.21151
HOXA9	0	17	0	0.016773	−0.51877	−0.29799
HSPA1B	0	17	0	0.033585	−0.30202	−0.19061
MCM7	0	17	0	0.034351	0.306314	0.120762
RING1	0	17	0	0.093199	0.582298	0.188265
STK33	0	16	0	0	−0.56107	−0.32061
GPX5	7552	16	0	4E-07	−0.47228	−0.37891
HMGB3	0	16	0	0.000632	0.382363	0.219011
ETV5	0	16	0	0.00449	−0.46575	−0.22203
NRM	0	16	0	0.012582	0.436479	0.293129
RPP30	0	16	0	0.022319	−0.26446	−0.1109
KDELC1	0	16	0	0.022657	−0.46574	−0.28722
RAB3C	0	16	0	0.027497	−0.44034	−0.28903
ZIC1	417	16	0	0.141174	−0.43631	−0.25775
TWIST1	338	16	0	0.296349	−0.45502	−0.22557
ATAT1	0	16	0	0.820333	0.65639	0.102174
ZNF433	0	15	0	1E-07	−0.59905	−0.37049
NMU	0	15	0	0.000407	−0.53554	−0.31553
C7orf50	0	15	0	0.000452	0.57173	0.285429
CXorf40B	0	15	0	0.001215	0.278825	0.179336
ZBED9	86509	15	0	0.003808	−0.48753	−0.33859
RPL10	0	15	0	0.006366	0.323866	0.214942
ZNF385B	0	15	0	0.008098	0.338216	0.22073
SNX32	0	15	0	0.010643	−0.4205	−0.26889
SLC35A2	0	15	0	0.021762	0.346339	0.230221
SRRM2	0	15	0	0.024278	0.817174	0.201275
H2AFY2	0	15	0	0.050995	−0.59059	−0.25905
C2	0	15	0	0.230738	0.503537	0.118451
PI4K2A	0	14	0	7.15E-05	−0.54557	−0.26069
EPB41L3	0	14	0	0.000249	−0.51336	−0.35133
AFF2	0	14	0	0.0004	0.378218	0.206431
ZNF860	0	14	0	0.001322	0.649719	0.229315
HOXB7	0	14	0	0.002277	−0.46049	−0.26313
PLD6	0	14	0	0.002642	0.486223	0.113408
XIAP	0	14	0	0.003657	0.264868	0.186815
TMEM196	0	14	0	0.003661	−0.52156	−0.31198
SPNS1	0	14	0	0.004457	0.401348	0.213285
IMPACT	0	14	0	0.006178	−0.44425	−0.30634
RNF113A	0	14	0	0.0206	0.341225	0.190005
HNRNPH2	0	14	0	0.022295	0.269486	0.183923
CLIP4	0	14	0	0.032589	−0.60327	−0.2899
VAX2	0	14	0	0.051513	−0.48038	−0.30245
SFRP2	841	14	0	0.168864	−0.44214	−0.2495
Sep-06	0	13	0	6E-07	0.556183	0.286346
KAZALD1	0	13	0	1.85E-05	0.650114	0.31254
KDM2B	0	13	0	2.44E-05	0.496711	0.354965
CYB5A	0	13	0	3.93E-05	−0.47664	−0.32126
DAXX	0	13	0	3.95E-05	0.541966	0.289617
ZNF132	0	13	0	4.39E-05	−0.50705	−0.29509
SLFN12	0	13	0	0.000194	−0.5644	−0.3652
ACY3	0	13	0	0.000353	0.406429	0.301805
ADAMTS19	0	13	0	0.000761	−0.53744	−0.36576
GUCY1A3	0	13	0	0.001093	−0.58692	−0.34127
ZNF793	0	13	0	0.002818	−0.47945	−0.36167
SSR4	0	13	0	0.004499	0.320181	0.226085
KCNH4	0	13	0	0.00665	−0.48712	−0.17119
STARD3NL	0	13	0	0.010012	−0.36683	−0.16709
UBE2A	0	13	0	0.012746	0.344197	0.235869
HOXA4	0	13	0	0.022408	−0.44749	−0.33147
THRB	0	13	0	0.05618	−0.47371	−0.31365
INS-IGF2	0	13	0	0.156887	−0.4652	−0.2835
DDX39B	0	13	0	0.175822	0.668181	0.236343
LCA5	0	12	0	5.8E-06	−0.64675	−0.4243
ZNF141	0	12	0	0.000405	−0.34543	−0.27028
DPYSL4	0	12	0	0.000656	−0.46166	−0.32116
CXCR5	0	12	0	0.002197	0.551853	0.25905
REC8	0	12	0	0.003484	−0.41665	−0.29553
AMMECR1	0	12	0	0.004318	0.32896	0.257902
WBSCR27	0	12	0	0.005331	−0.46873	−0.18978
RRAS2	0	12	0	0.005459	−0.38537	−0.18109
ZNF449	0	12	0	0.006875	0.248954	0.197997
LRRC14B	0	12	0	0.007127	0.511213	0.241903
DLX5	0	12	0	0.023855	−0.45667	−0.27103
MMP2	0	12	0	0.062498	−0.48527	−0.27952

Top 100 most significant DMRs are shown (FDR < 0.05, no.probes > 1), sorted by the number of CpGs per region.

To gain insights into the biological effect of altered DNA methylation patterns in EBV (+) BL, we performed pathway analysis of genes localised within or in proximity of the DMR (Fig. 3B,C). Hypomethylated regions were enriched in pathways such as tight junction, cGMP-PKC pathway and other pathways deregulated in cancer (Fig. 3B). In addition, they were frequently localized to B cell binding sites for polycomb 2 enzymes (such as SUZ12 and EZH2) and enriched for H3K27me3 histone marks, as assessed by the ENCODE Transcription Factor and Histone Modifications (Fig. 3B), therefore at genomic sites which are normally heterochromatic. A different pattern of enrichment was observed for the hypermethylated regions, with top pathways including B cells receptor, NFκB and Notch1 signalling pathways. Hypermethylated regions were also enriched for RUNX1 binding sites and co-localize with regulatory and active histone marks (H3K4me1+, H3K36me3+) (Fig. 3C). This last observation is in line with our results showing that hypermethylated sites tended to be localised at enhancer regions (Fig. 2B–D and F).

### Cancer related genes are epigenetically silenced in eBL derived cell lines

**Figure 4:**
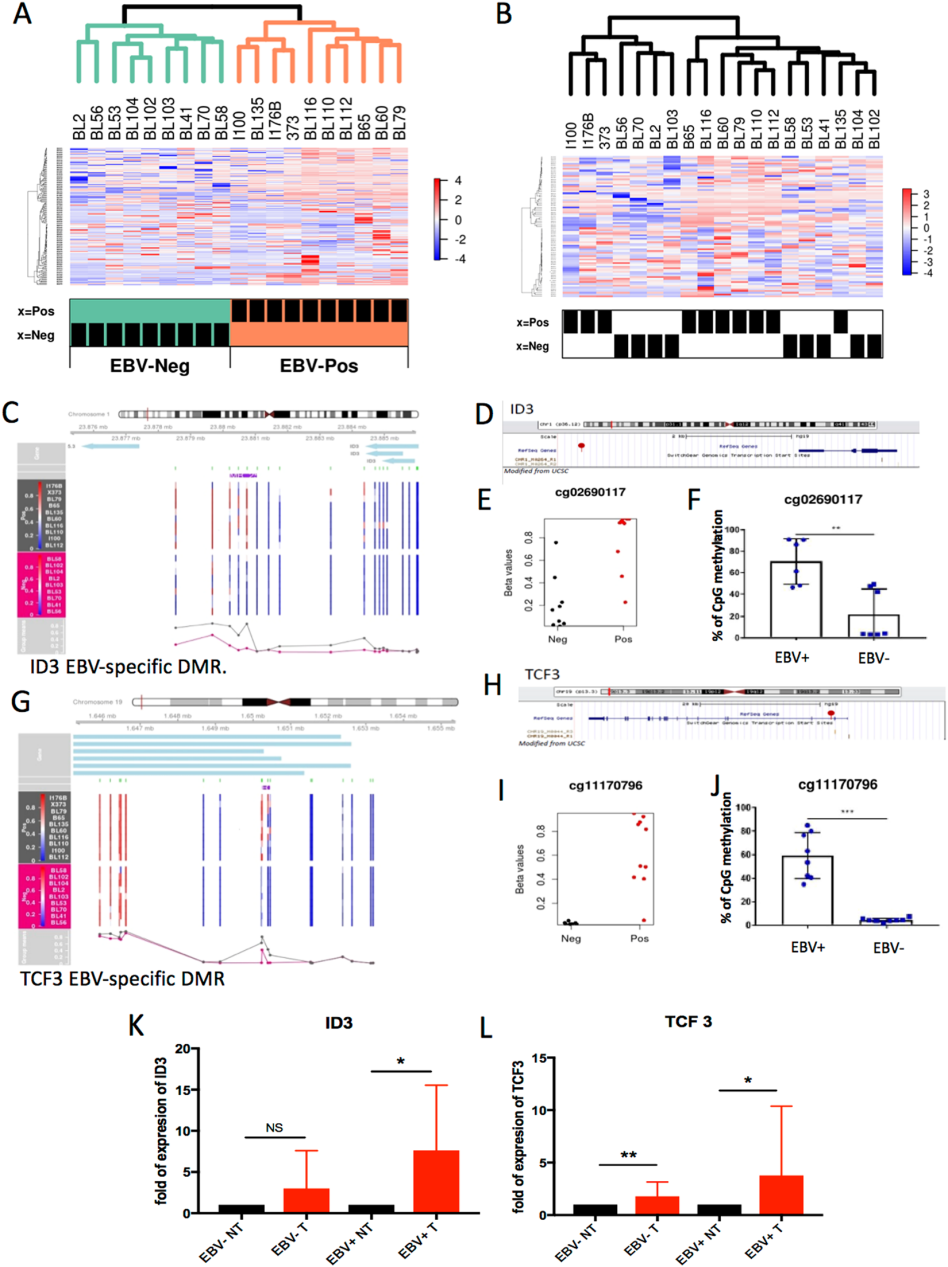
Unsupervised clustering of sBL or eBL mutational signatures in EBV (+) and EBV (−) BL. Methylation of sBL drivers in EBV (+) and EBV (−) BL cell lines (**A**). Methylation of eBL drivers in EBV (+) and EBV (−) BL cell lines (**B**). (**C** and **G**) DMR plot corresponding to ID3 and TCF3 regions. (**D** and **H**) Schematic representation of ID3 promoter, red dots identify cpg of interest (adapted from UGC web site). (**E** and **I**) strip-chart plots showing differential methylation in EBV pos and EBV neg BLs at position cg02978140 (**E**) and cg11170796 (**I**), on ID3 and TCF3 promoters respectively. (**F** and **J**) The histograms show the average % of methylation of cg02978140 (**F**) and cg11170796 (**J**) in the DNA of 7 EBV (+) and 7 EBV (−) BLs, measured by pyrosequencing (**p value < 0.01, ***p value < 0.001). (**K** and **L**) Three EBV (+) and 3 EBV (−) BL cell lines were cultured in presence of 5-Aza-2′-deoxycytidine at the final concentration of 10 μM for 48 h (T = treated) or with DMSO (NT = untreated). mRNA levels of ID3 and TCF3 were analyzed by qPCR. The pooled results of three independent Aza treatment are represented in the histograms (*p value < 0.05, **p value < 0.01, ns = non-significant).

A recent study interrogated the mutational landscape of endemic BL (eBL)^14^ and revealed lower frequencies of mutations in ID3 and TCF3 in the endemic BL variant compared to the sporadic variant. Therefore, we examined whether in eBL, EBV may target mutational drivers by epigenetic silencing. To this end, we first drew heat-maps for each of the driver subsets, sBL (e.g. MYC, ID3, TCF3, and TP53) and eBL (e.g. ARID1A, RHOA, and CCNF). Our BL samples were hierarchically classified according to EBV status when selecting the sBL mutational signature (Fig. 4A), while this was not the case with the known eBL driver genes (Fig. 4B). Indeed, two of the DMRs identified by comparing whole methylome profiles of EBV (+) and EBV (−) BL cell lines mapped to two genes frequently mutated in sBL: ID3 and TCF3 (Fig. 4C and G). Moreover, two specific CpG positions within ID3 and TCF3 promoters (Fig. 4D and H) appeared to be differentially methylated in EBV (+) and EBV (−) BL cell lines (Fig. 4E and I). In line with the significant difference in the levels of CpG methylation, validated by direct pyrosequencing (Fig. 4F and J), ID3 and TCF3 expression levels were low or undetectable in the majority of the analyzed EBV (+) BL compared to the EBV (−) BL derived cell lines and primary B cells (Table 3). To assess if DNA methylation played a role in regulating the expression of ID3 and TCF3 in BL, we treated 3 EBV (+) and 3 EBV (−) BL cell lines with the demethylation agent 5-Aza-2′-deoxycytidine (Aza). Demethylation of the DNA by Aza treatment led to rescue of the expression levels of ID3 in all the EBV+ cell lines (Fig. 4K), whereas no significant ID3 mRNA changes were observed in EBV− BLs. This finding indicates that DNA methylation modulates ID3 expression levels in BL EBV+ cell lines. Upon Aza treatment, TCF3 expression levels also increased significantly in EBV+ BLs; a lower but significant increase of TCF3 mRNA was observed in Aza-treated EBV (−) BL derived cell lines (Fig. 4L).

**Table 3:** mRNA levels of ID3 and TCF3 in EBV (+) and (−) BL cells and primary B cells.

		TCF3	ID3
EBV+	I176	0	0
	I100	2.142857	0
	BL 65 2	4.016949	0.864407
	BL 79	0	0
	BL 135	0	0
	BL 60 1	0	0
EBV−	BL 41 3	0	0
	BL 53 3	9.75	0
	BL 70 2	5.625	0.910714
	BL 103	0.906977	1.238372
	BL 102 2	3.169014	2.15493
	BL 104 2	1.273469	1.297959
primary	Don.1	2.213793	1.17931
	Don.2	1.888235	0.723529
	Don.3	3.178218	2.049505
	Don.4	1	1

The analysis of the methylome profiles of EBV (+) and EBV (−) BL derived cell lines led to identification of other genes with a potential role in transformation (such as RRSA, KDM2B, TGFB1 or IGFB1) and that could be differentially regulated in the two groups of BL. Q PCR analysis of the mRNA levels of these genes showed that they were significantly down regulated in all the analyzed BLs (14 lines) compared to primary B cells (from 4 independent healthy donors) independently of the EBV status (Supplementary Fig. 2A), however DNA demethylation by Aza treatment rescued their expression only in EBV+ BLs (Supplementary Fig. 2B). This observation indicates that BLs have similar gene expression patterns independently of the EBV status, however the mechanisms of gene expression regulation are different. Indeed, comparative analysis of the RNA profiles of 5 EBV+ and 5 EBV− BL cell lines performed by illumina RNA array showed that, despite the great difference observed in the pattern of methylation, the two groups of BLs did not differ significantly in whole-genome expression profiles (Supplementary Fig. S1A), with the exception of a subset of genes, in part involved in immunity and metabolic pathways (Supplementary Fig. S1B and Table S2). Together, our results identified known and potential drivers in lymphomagenesis which are epigenetically silenced in EBV+ BL cell lines.

### EBV infection of B cells induces epigenetic silencing of ID3

**Figure 5:**
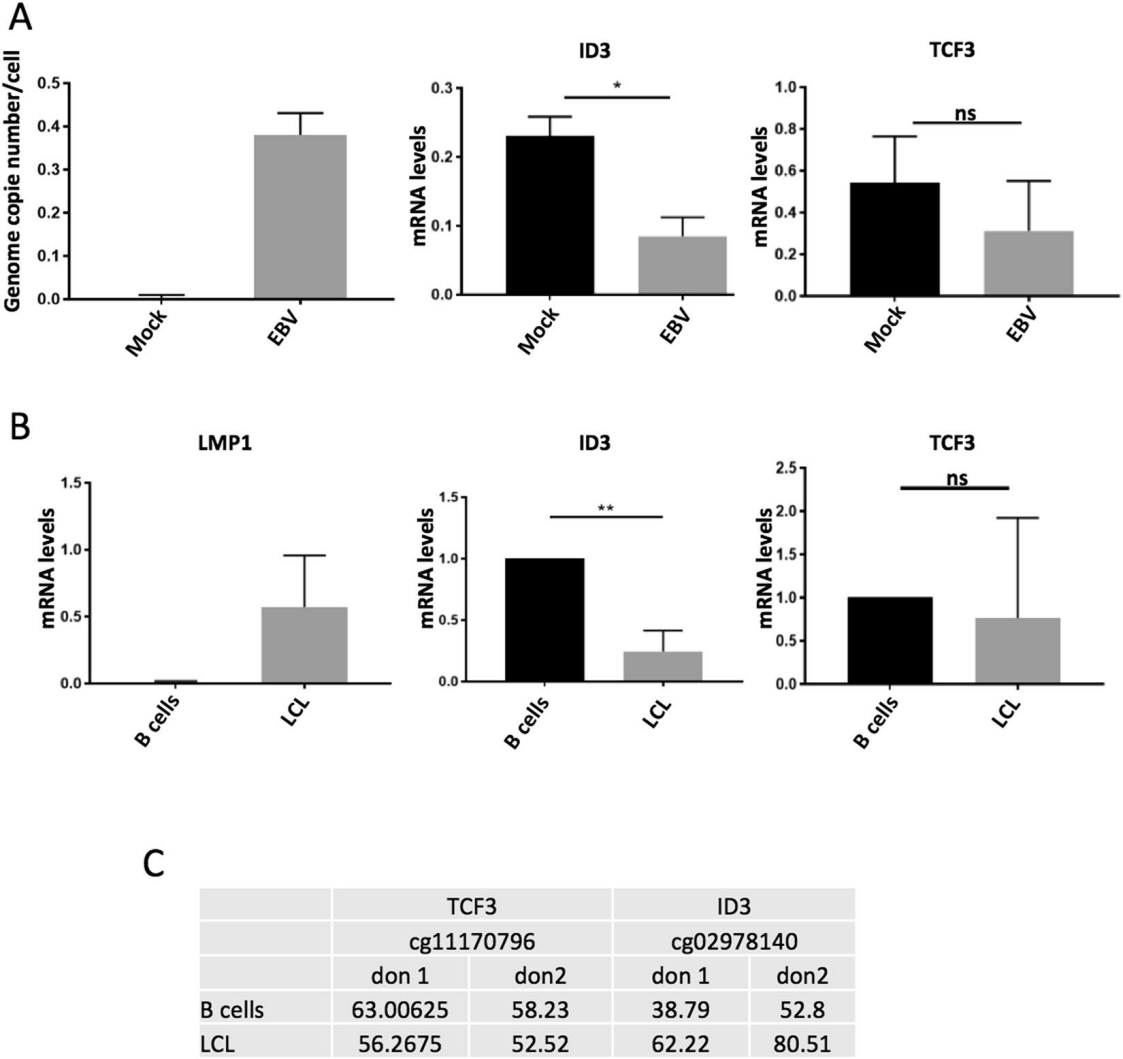
EBV-dependent silencing of ID3 expression *in vitro*. (**A**) Loucks cells were infected with EBV or mock infected for 48 h, then collected and processed for RNA/DNA extraction. 100ng of DNA were analysed by Taqman PCR for the EBV genome copy number (right panel). Total RNA was retro-transcribed and cDNA analysed by qPCR for the levels of ID3 and TCF3 (central and left panel). The histograms show the average results of two independent infections (*p value < 0.05; ns = non-significant). (**B**) Primary B cells from three different donors were putted in culture for 24–36 h, after that cells were in part collected to make dry pellets and in part infected with EBV and cultured until they got immortalized (LCL). Nucleic acids were extracted from primary and immortalized cells, total RNA retro-transcribed and analyzed by qPCR for the levels of the indicated genes. Histograms show the average mRNA levels for the indicated genes, measured in B cells and in the corresponding LCL from three independent donors (**p value < 0.01; ns = non-significant). (**C**) DNA from primary and immortalized matched samples from 2 donors were processed for pyrosequencing and analysed for the levels of methylation of the cg02978140 and cg11170796 positions.

Despite the key role of ID3 and TCF3 in the pathogenesis of BL, no previous studies have investigated if during EBV infection of B cells the virus can directly modulate their mRNA levels. To test this possibility, we infected Louckes, an EBV negative BL cell line, with EBV and analyzed the cells for EBV gene copy number, as well as for levels of ID3 and TCF3 mRNAs (Fig. 5A). We also studied EBV-infected primary B cells from different donors, allowed them to grow until immortalized (LCL) and then compared each LCL to primary B cells from the corresponding donor for the mRNA levels of ID3 and TCF3 (Fig. 5B). In line with the observation that the promoter of ID3 is highly methylated in EBV+ BL, EBV infected B cells showed reduced levels of the ID3 transcript (Fig. 5A and B), consistent with a direct effect of EBV on the regulation of ID3 mRNA levels. Similar results were obtained by infecting RPMI-8226, an EBV (−) myeloma-derived cell line (data not shown). No significant difference in the mRNA levels of TCF3 was observed upon EBV infection of immortalized B cells, nor in LCL compared to primary B cells (Fig. 5A and B). In agreement with these results, by pyrosequencing we found increased methylation levels of the cg02978140 at the ID3 promoter in EBV-immortalized B cells compared to the corresponding primary B cells (from two independent donors), however we did not observe changes in methylation levels of cg11170796 on the TCF3 promoter (Fig. 5C).

### EBV induces epigenetic silencing of ID3 via LMP1

**Figure 6:**
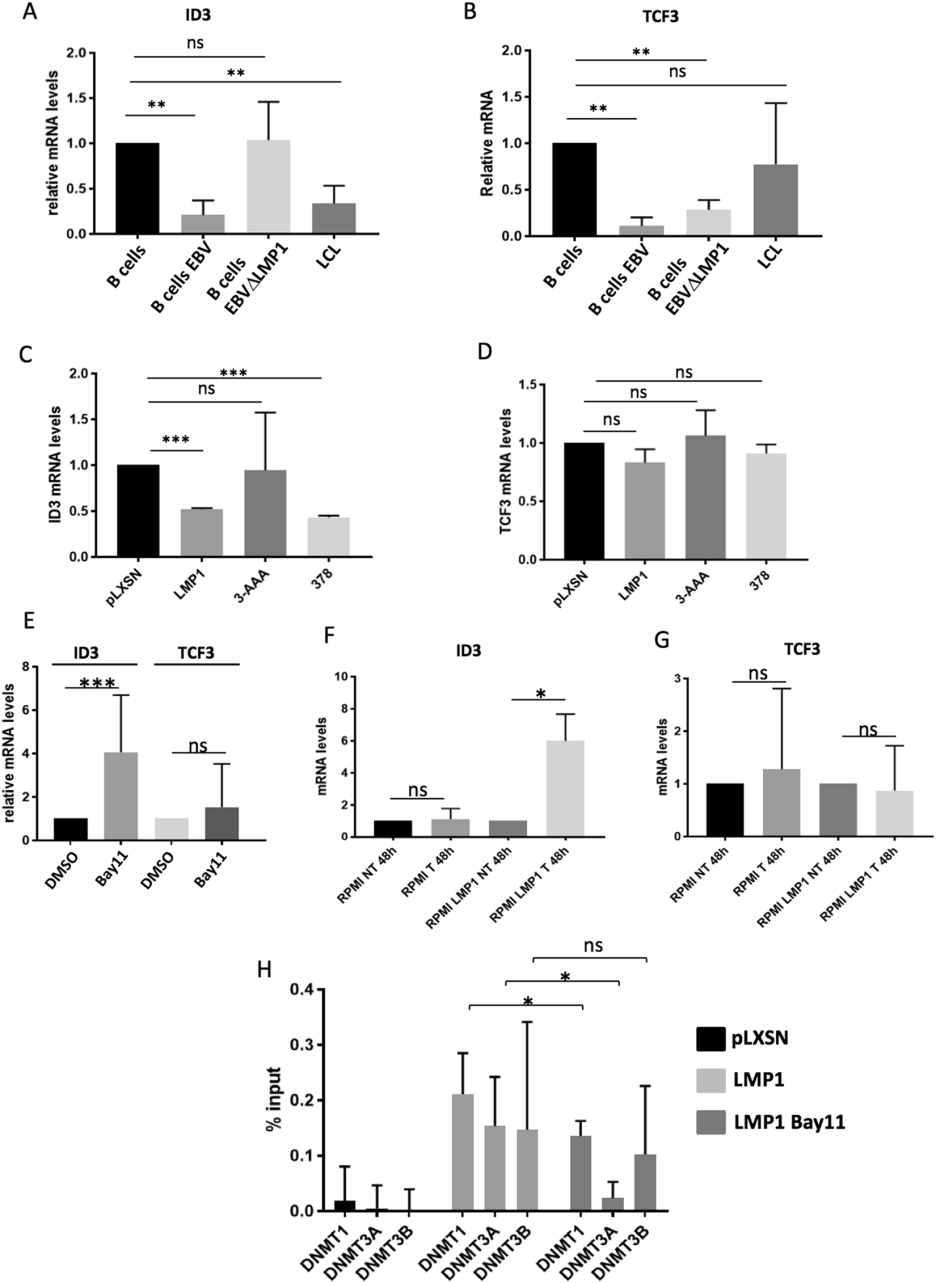
LMP1-mediated downregulation of ID3. (**A** and **B**) B cells from two donors were infected with WT or ΔLMP1 EBV and collected 48 h post-infection. Retro-transcribed RNA samples were analyzed by qPCR for the levels of ID3 (**A**) or TCF3 (**B**) (**p value < 0.01). (**C** and **D**) RPMI cells were stably transduced with pLXSN (pLXSN) or with pLXSN-LMP1 WT (LMP1) or mutated (3-AAA and 378). cDNA samples were interrogated by qPCR for the levels of ID3 (**C**) and TCF3 (**D**) (***p value < 0.001). (**E**) RPMI-LMP1 cells were treated for 2 h with Bay11 (10 μM). cDNA samples were analyzed by qPCR for the mRNA levels of ID3 and TCF3 (***p value < 0.001). (**F** and **G**) RPMI or RPMI-LMP1 cells were cultured in presence of Aza (T) or in DMSO (NT) and the mRNA levels of ID3 and TCF3 were analyzed by qPCR (***p value < 0.001). (**H**) RPMI pLXSN and RPMI-LMP1 cells, the latter, treated or not with Bay11 (10 μM) for 2 h, were used to perform ChIP with indicated antibodies. The eluted DNA was analyzed by qPCR with primers designed in the promoters of ID3 (*p value < 0.05).

To further characterize the mechanism whereby EBV downregulates the expression of ID3, we aimed to determine which EBV protein played a role in this event. The Latent membrane protein (LMP1) is well known to contribute to EBV-associated oncogenesis. Therefore we infected primary B cells from 2 independent donors with WT or ΔLMP1 EBV genome. Two days after infection cells were collected and analyzed by qPCR for the levels of EBER and LMP1. WT and ΔLMP1 EBV infected B cells showed similar levels of EBER, indicating that the two infections worked with similar efficiency (Supplementary Fig. S3A). As expected, ΔLMP1 EBV infected B cells had undetectable LMP1 levels (Supplementary Fig. S3B). In line with the results obtained in EBV infected Louckes and RPMI-8226, primary B cells infected with WT EBV showed a significant downregulation of ID3 mRNA levels that was also maintained after immortalization, in the corresponding LCL (Fig. 6A). Infection of B cells with ΔLMP1 EBV genome, conducted in the same experimental condition, let unchanged the ID3 mRNA; meaning that LMP1 is playing the major role in this event. By contrast, infection of B cells with both WT and ΔLMP1 EBV genomes led to the downregulation of TCF3 mRNA (Fig. 6B), which indicates that this event is independent of LMP1. In line with other experiments (Fig. 5B), EBV immortalized B cells (LCL) showed similar levels of TCF3 mRNA compared to the parental primary B cells (Fig. 6B). Next we analyzed ID3 and TCF3 mRNA levels in RPMI-8226 cells stably expressing LMP1. The EBV transforming protein alone was able to inhibit ID3 expression, but did not change TCF3 mRNA levels (Fig. 6C and D, compare first and second lines). However, stable expression in RPMI cells of an LMP1 mutated in the C-terminal activation region 1 (CTAR1) (amino acids 187–231), that has lost the ability of activating NFκB pathway, did not affect the levels of ID3 when compared to the RPMI-pLXSN control cells (Fig. 6C, compare first and third lines). On the contrary, CTAR2 LMP-1 mutant (LMP-1/378 stop), that is unable to activate JNK-1, retained the ability to downregulate ID3 (Fig. 6C, compare first and fourth lines). Again, neither WT LMP1 nor LMP1 mutants affected the levels of TCF3 when stably expressed in RPMI cells (Fig. 6D). The WT and LMP1 mutants were expressed at comparable levels in the retro-transduced RPMI (Supplementary Fig. S3C). To confirm that LMP1-mediated deregulation of ID3 requires activation of NFκB pathway we treated RPMI-LMP1 cells with the compound BAY 11-7082, a chemical inhibitor of IκBα phosphorylation and degradation, largely used to block the canonical NFκB pathway. In line with the results obtained with the LMP1 mutants, ID3, but not TCF3 mRNA levels were significantly rescued when activation of the NFκB pathway was hampered by the BAY 11-7082 treatment (Fig. 6E). As, our data show that the EBV-mediated downregulation of ID3 occurs by methylation of the gene promoter, we next asked whether LMP1 also alters ID3 expression by triggering epigenetic changes on the ID3 promoter. Indeed, treating RPMI-LMP1 cells with the DNA demethylating agent, Aza, led to a significant rescue of ID3 levels (Fig. 6F), but left unchanged the levels of TCF3 (Fig. 6G), indicating that DNMTs could play a role in LMP1-mediated inhibition of ID3. We therefore performed ChIP-qPCR experiments in RPMI -pLXSN or RPMI -LMP1 cells to quantify the amount of DNMT1, 3A and 3B recruited onto ID3 regulatory promoter regions, in the proximity of the cg02978140. None of the three DNMTs bound to this region in RPMI pLXSN, but they were efficiently recruited to it in the presence of LMP1 (Fig. 6H). Interestingly, we observed a significant reduction of DNMT1 and DNMT3A recruitment to the ID3 promoter, in BAY 11-7082 treated RPMI LMP1 (Fig. 6H). Taken together, these data indicate that EBV induces epigenetic silencing of ID3 expression *via* its main transforming protein LMP1 and suggest that LMP1 triggers the recruitment of the DNMTs on the promoter of ID3, an activity that is in part mediated by its ability to activate NFκB.

## Discussion

Fifty years after the discovery of EBV particles in eBL derived cultures, enough evidence has been cumulated to establish a causal relationship between EBV infection and BL, but the mechanistic role of the virus in the carcinogenic process remains to be elucidated.

Previous studies have focused on the characterization of the genetic landscape of the different BL clinical variants^10, 20, 21^. Recently, Abate and collaborators have shown that eBL have a lower mutation burden than the sporadic clinical variant^14^. We therefore hypothesized that a virus-mediated mechanism could alter cellular expression and release the selective pressure for the accumulation of driver gene mutations. Epigenetic silencing of tumor suppressors has been shown in several cancers, including NPC and EBVaGC^22–26^. Moreover viral-driven epigenetic changes have been shown to be involved in the EBV-mediated repression of individual tumor suppressor genes, such as Bim1^27^ or Blimp1^28^ and contribute to the process of lymphomagenesis. Some studies have also shown epigenetic changes in sporadic BL^29–31^. However, to our knowledge only one study has compared the whole methylation and mutational profile in one endemic EBV+ BL derived cell line, DAUDI^32^. In the current work comparing whole methylome profiles in a panel of EBV+ vs EBV− BL derived cell lines, we found a clear EBV epigenetic signature. Differentially methylated positions in EBV+ BL vs EBV− BL were located in close proximity to genes involved in pathways with a role in B cell function and development (such as BRC receptor and Notch pathway) but often altered in lymphoproliferative disease^33–35^. Moreover many of the EBV associated DMPs were found in proximity to SUZ and EZH2 binding sites in B cells. This is relevant considering that deregulation of EZH2 activity, due to gain of function mutations, is a key event in lymphomagenesis^36, 37^. One could speculate that the changes of DNA methylation levels in proximity to the EZH2 binding region observed in EBV+ BLs, could be due to virus-induced changes in the amount or activity of PC2 enzymes and/or of DNMT1 that is known to interact with EZH2^38^. Furthermore, we observed that in EBV+ BLs, the hypomethylated DMPs were common in promoter regions (0–1 kb from transcriptional start sites). On the contrary, hypermethylated sites were in average significantly more distant from TSS, with a trend to be enriched in enhancer regions. This different scenario between EBV (+) and (−) BLs, could also be the consequence of the viral-mediated deregulation or redistribution of the epigenetic modifiers. The biological meaning of this hypermethylation at the enhancer sites will be the object of further investigations.

To assess whether EBV-associated differential methylation of specific gene sites led to altered gene expression and to gain insight into the biological relevance of the identified epigenetic changes, we analyzed the RNA levels of a panel of genes in proximity to DMPs, in EBV (+) and EBV (−) BL cell lines compared to primary B cells from different donors. Some genes were differentially expressed in the two BL groups and appear to be regulated by DNA methylation in EBV (+) BLs. Among them, we found ID3 and TCF3 often mutated in sporadic BL^10, 14^. For the first time we show that EBV infection in B cells leads to increased methylation of ID3 promoter and silencing of ID3 expression by a LMP1-mediated mechanism. LMP1 is known to modulate the levels of DNMTs in GC B cells^39^, indeed our data indicate that during EBV infection LMP1 increases the recruitment of the DNMTs to the promoter of ID3; this activity of LMP1, that appears to require its ability to induce NFκB, could then result in increased methylation of ID3 promoter and silencing of its gene expression; further studies are needed to validate this hypothesis.

Despite the strong difference in levels of methylation of TCF3 promoter in EBV (+) BL versus EBV (−) BL cell lines, TCF3 mRNA levels are not directly altered by EBV infection or expression of LMP1 *in vitro*. This implies that additional events may occur *in vivo* to regulate TCF3 levels, possibly independent of the presence of EBV, as supported by the observation that DNA demethylation by Aza leads to a significant rescue of TCF3 mRNA levels in both EBV(+) and EBV(−) BLs; this also indicates that methylation at different CpG positions could be one of the mechanisms responsible for TCF3 deregulated expression in EBV− BL, additional to the frequent mutation rate reported in sporadic BL. In line with an epigenetic modulation of the ID3-TCF3 axes in EBV (+) BLs, we also found DMRs in proximity of ID2 and TWIST1; the latter is known to interact with TCF3 in presence of low levels of ID proteins^40, 41^. We also found other genes differentially methylated and expressed in EBV (+) BLs that were not previously associated with endemic BL, but that are known to play a role in many cancers. This includes RUNX1 (data not shown), known to be involved in B cell and lymphoid development, and whose deregulation can accelerate Myc-induced lymphomagenesis^42^, or the lysine (K)-specific demethylase 2B (KDM2B), an important mediator of hematopoietic cell development that has opposing roles in tumor progression depending on the cellular contest^43^. Altered activity of different KDMs has been associated with cellular transformation. Anderton and collaborators reported the EBV-mediated deregulation of KDM6B and its role in Hodgkin’s lymphoma, however no previous study has assessed the role of KDM2B in the process of EBV-mediated B cells transformation. It has been shown that recognition of demethylated CpG island by KDM2B targets them for polycomb-mediated silencing^44^. Altered levels of KDM2B could then affect both host and virus chromatin structure and gene expression. Therefore further functional characterization of KDM2B promoter methylation in EBV+ BLs is warranted. DNA demethylation by Aza treatment and direct pyrosequencing confirmed the EBV-mediated epigenetic regulation of these genes.

Some of the analyzed genes appeared to be repressed in all BL cell lines when compared to primary B cells, independently of the EBV status. This was confirmed by whole expression profiles in EBV (+) and (−) BLs, which showed that despite the profound difference in their epigenetic profiles, EBV (+) and (−) BLs appear to be phenotypically very similar as they exhibited few differences in RNA levels. In our view this result confirms that similar events occur in the process of lymphomagenesis, but the molecular mechanisms and the selective pressure through which the cells pass during BL pathogenesis are different in the viral and non-viral-BL variants. Future studies will be needed to assess if these methylation patterns identified in BL-derived cell lines are also found in BL *ex vivo* samples.

In summary, this study describes the methylome signatures and the expression profile of different EBV (+) and EBV (−) BL derived cell lines and shows the EBV-mediated epigenetic silencing of drivers in B cell transformation therefore demonstrating an active role of the virus in the process of lymphomagenesis.

## Materials and Methods

### Cell culture

Peripheral B cells were purified from blood samples as previously described^45^. The myeloma-derived RPMI-8226 cells (http://web.expasy.org/cellosaurus/CVCL_0014) and the Burkitt lymphomas cell lines (BL), including the BL EBV(−) cell line Louckes (http://web.expasy.org/cellosaurus/CVCL_8259), were obtained from the IARC biobank. The EBV genome copy number determined by Taqman PCR and the geographical origin of BL used in the present study are described in Supplementary Table 1. Primary and immortalized B cells were cultured in RPMI 1640 medium (GIBCO; Invitrogen life Technologies, Cergy-Pontoise, France) supplemented with 10% FBS, 100 U/ml penicillin G, 100 mg/ml streptomycin, 2 mM L-glutamine, and 1 mM sodium pyruvate (PAA, Pasching, Austria) or in advanced RPMI 1640 (LIFE TECHNOLOGIES; 12633012). EBV (Akata strain) particles produced by culturing Hone-1 EBV cells were used to infect B cells. EBV infections of B cells were performed either using WT EBV genome or using a EBV strain lacking the entire LMP-1 gene (EBVΔLMP-1).

To demethylate the DNA cells were treated with 5-Aza-2′-deoxycytidine ≥97%, (Sigma Aldrich; A3656) at the final concentration of 10 μM for 48 h and/or 96 h. To inhibit the canonical NFκB pathway, cells were treated with the IκBα kinase inhibitor Bay11-7082 (10–20 μM) (Calbiochem) for 2 h.

### RT-PCR and Quantitative PCR

Extraction of total RNA, reverse transcription to cDNA and quantitative PCR (qPCR) were performed as previously described^45^. For each primer set the qPCR was performed in duplicate and the mRNA levels obtained were normalized on the average mRNA levels of three housekeeping genes (β-globine, β-actine, GAPDH), measured in the same samples.

EBV genome copy number per cell was measured by Taqman PCR, primers and probes described in Accardi *et al*.^45^. The PCR primer sequences are indicated in Supplementary Table 3.

### Chromatin immunoprecipitation

Chromatin immunoprecipitation (ChIP) was performed with Diagenode Shearing ChIP and OneDay ChIP kits according to the manufacturer’s protocols, by using the following antibodies: DNMT1 (Abnova MAB0079), DNMT3A (Abcam ab13888), DNMT3B (Abcam ab13604) and IgG (Diogenode). The eluted DNA was used as template for qPCR with primers designed on the promoter region of ID3 (5′-GCCACTGACTGACCCCTAAG-3′ and 5′-CCCGGTTCCTTCCTTCCTT-3′).

### Bisulfite modification and pyrosequencing

Cells were pelleted and resuspended in lysis buffer (1% SDS, 0.1 M NaCl, 0.1 M EDTA, 0.05 M Tris pH8) with Proteinase K (500ug/ml) and incubated for 2 hours at 55 °C. DNA was saturated with NaCl (6 M), precipitated with isopropanol, and cleaned with 70% ethanol. Extracted DNA was finally resuspended in water. Quantity and quality of the extracted DNA were assessed with a ND-8000 spectrophotometer (Nanodrop, Thermo scientific). To quantify the percentage of methylated cytosine in individual CpG sites, we performed bisulfite pyrosequencing, as previously described^46^. For samples processed with Illumina 450 K Infinium bead arrays (see below), 600 ng of DNA was converted using the EZ DNA methylation Kit (Zymo Research) and modified DNA was eluted in 16 ul of water. Quality of modification was checked by PCR using modified and unmodified primers for *GAPDH* gene.

### Bead array methylation assays

Methylation profiles of the different samples were analyzed using the 450 K Infinium methylation bead arrays (Illumina, San Diego, USA). Briefly, the Infinium Humanmethylation450 beadchip interrogates more than 480,000 methylation sites^47^. The analysis on the bead array was conducted following the recommended protocols for amplification, labelling, hybridization and scanning.

### Bioinformatics Analysis

Raw methylation data was imported and processed using R/Bioconductor packages^48, 49^. Data quality was inspected using boxplots for the distribution of methylated and unmethylated signals, and inter-sample relationship using multidimensional scaling plots and unsupervised clustering. Probes were filtered for low quality (detection P value > 0.05) and known cross-reactive probes^50^. The remaining dataset was background subtracted, and normalized using intra-array beta-mixture quantile normalization^51^. Methylation beta values were logarithmically transformed to M values before parametric statistical analyses, as recommended^52^. To define differentially methylated positions (DMPs) and differentially methylated regions (DMRs), we modelled the EBV status as a categorical variable in a linear regression using an empirical Bayesian approach^53^. DMPs were selected based on a differential methylation (delta beta) of at least 40% when comparing the two EBV categories. DMRs were identified with the DMRcate package using the recommended proximity-based criteria^54^. A DMR was defined by the presence of at least 2 differentially methylated CpG sites with a maximum gap of 1000 bp. Differentially methylated genes (DMPs and DMRs) were further analyzed to determine functional pathways and ontology enrichment using Enrichr^55^. All methylation data have been deposited to the Gene Expression Omnibus repository (GEO accession number GSE92378).

### Whole Genome Expression Analysis

Differential expression analysis was performed using Human HT-12 Expression BeadChips (Illumina) as previously described^45, 56^ and 500 ng total RNA isolated with the TRIzol Reagent (Invitrogen) according to the manufacturer’s instructions. Raw expression bead array data (AVG-Signal), with no normalization and no background subtraction was exported from Genome Studio (version 2011.1, Illumina) into BRB-ArrayTools software (version 4.3.1, developed by Dr. Richard Simon and the BRB-ArrayTools Development Team. Data were normalized and annotated using the R/Bioconductor package “lumi”^48^. Quality of the data was assessed by plotting the distribution of the intensity for all probes, and a correlation between technical replicates performed. Class comparison between groups of bead arrays was done computing a t-test separately for each gene using the normalized log-transformed beta values. Only those probes with p value < 0.01, false discovery rate (FDR) < 0.05 and a fold-change of at least 1.5 were considered differentially expressed.

### Statistical analysis

Statistical significance was determined by Student T test. The p value of each experiment is indicated in the corresponding Figure legend. Error bars in the graphs represent the standard deviation.

## Electronic supplementary material

supplementary figures and tables
