# Supplementary material for: Viral driven epigenetic events alter the expression of cancer-related genes in Epstein-Barr-virus naturally infected Burkitt lymphoma cell lines
Source: Sci Rep. 2017 Jul 19;7:5852. doi: 10.1038/s41598-017-05713-2 (PMC5517637; doi:10.1038/s41598-017-05713-2)
Supplement: Supplementary file 1 — supplementary figures and tables [file 41598_2017_5713_MOESM1_ESM.pdf]

Viral driven epigenetic events alter the expression of cancer-related genes in Epstein-Barr-virus naturally infected Burkitt lymphoma cell lines

Hector Hernandez-Vargas, Henri Gruffat, Marie Pierre Cros, Audrey Diederichs, Cécilia Sirand, Romina C. Vargas-Ayala, Antonin Jay, Geoffroy Durand, Florence Le Calvez-Kelm, Zdenko Herceg, Evelyne Manet, Christopher P. Wild, Massimo Tommasino, Rosita Accardi

1SA

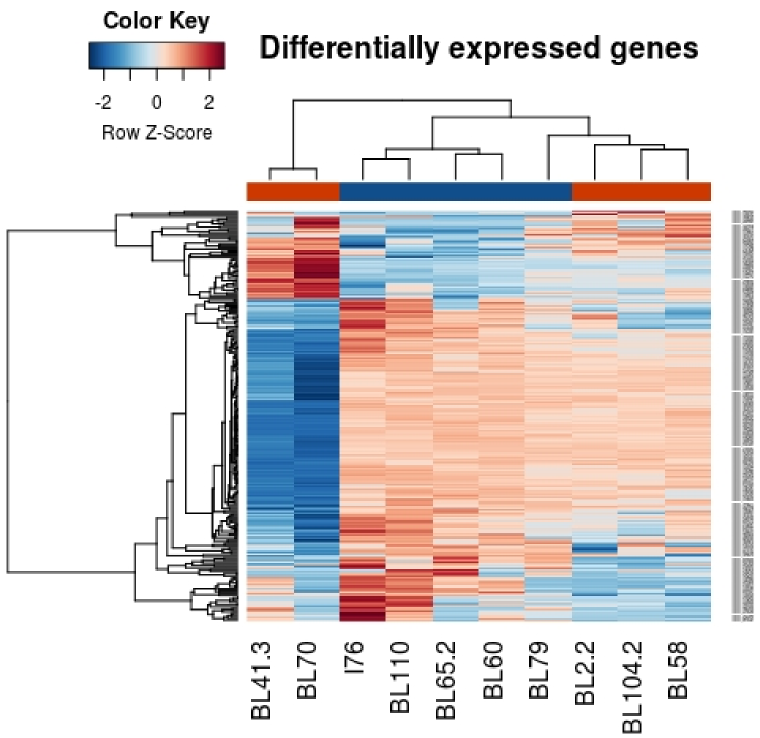

1SB

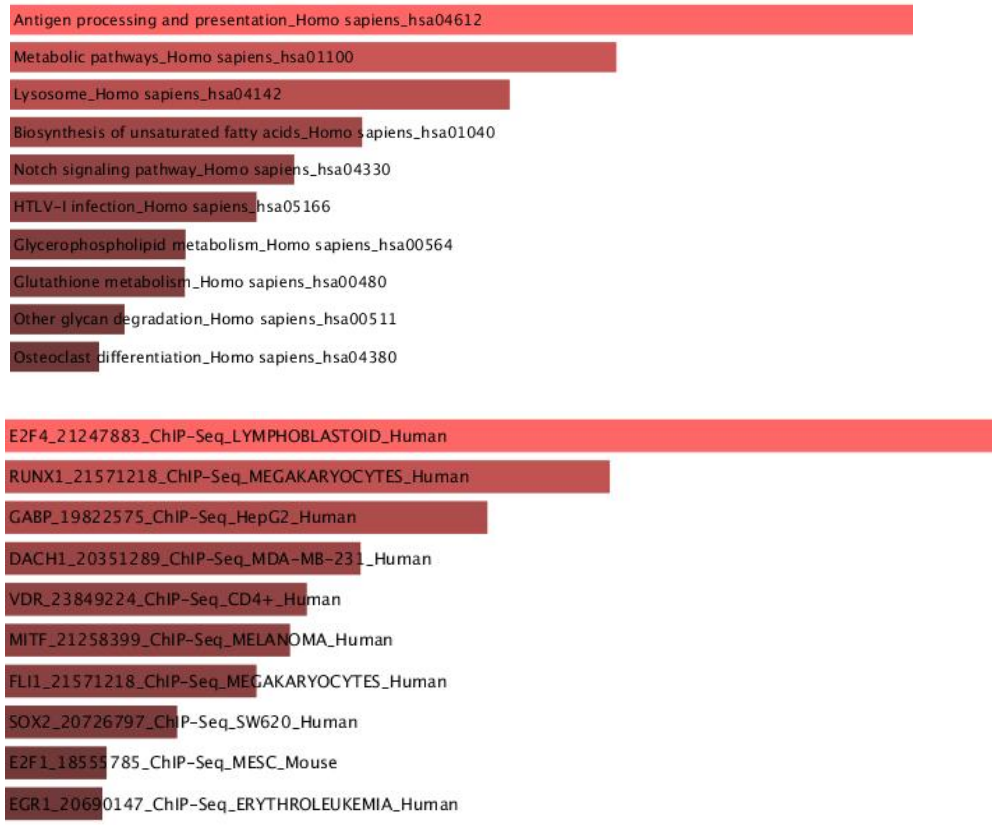

Supplementary Fig 1. 1SA) Heat map of genes differentially regulated in 5 EBV (+) vs. 5 EBV (-) BL. **1SB)** to identified pathways enriched of differentially expressed genes Enrich web tool was used. Enrichment results for KEGG database are shown.

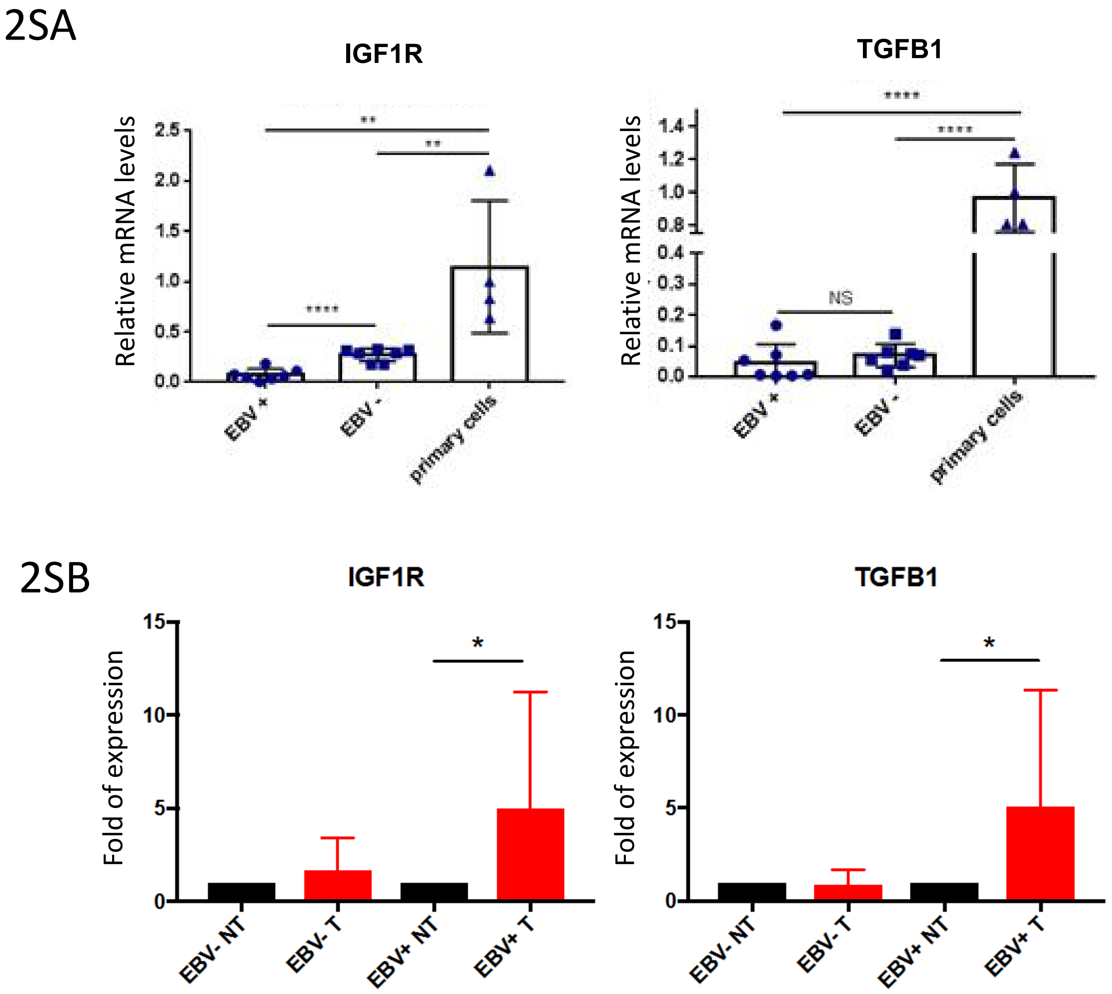

Supplementary Fig 2: 2SA) mRNA levels of IGF1R and TGFB1 were measured in 7 EBV (+) , 7 (-) BL cell lines and in primary B cells (from 4 different donors) by qPCR using the following primers (IGF1R-Fw 5’ ACA ACT ACG CCC TGG TCA TC 3’; IGF1R Rev 5’ CAT TCC TTT GGG GGC TTA TT 3’; TGFB1-Fw 5’ GGG ACT ATC CAC CTG CAA GA 3’ and TGFB1-Rev 5’ CCT CCT TGG CGT AGT AGT CG 3’.). 2SB) Three EBV (+) and 3 EBV (-) BL were cultured in presence of 5-Aza-2’-deoxycytidine at the final concentration of 10mM for 48h (T=treated/red bars) or with DMSO (NT=untreated/black bars). mRNA levels of ID3 and TCF3 were measured by qPCR. The pooled results of three independent Aza treatment are represented in the histograms (*p value<0.05).

Supplementary figure 2 Hernandez et al. 2017

3SA

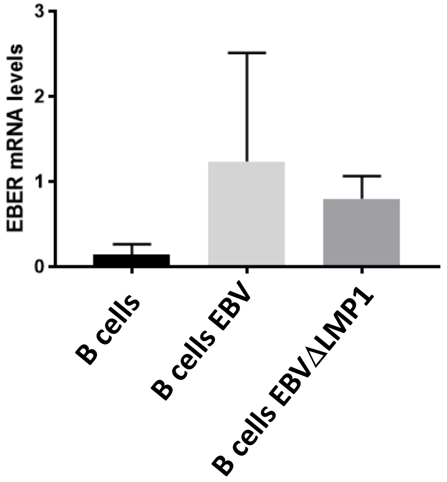

3SB

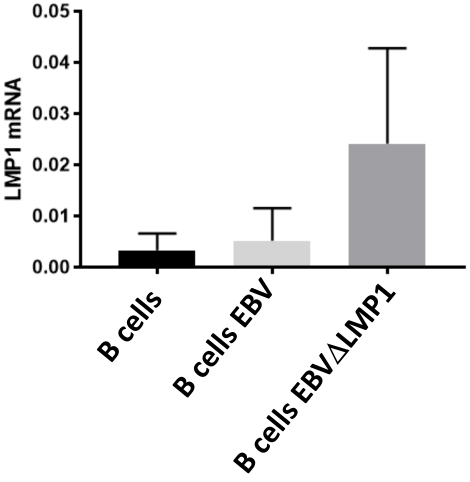

3SC

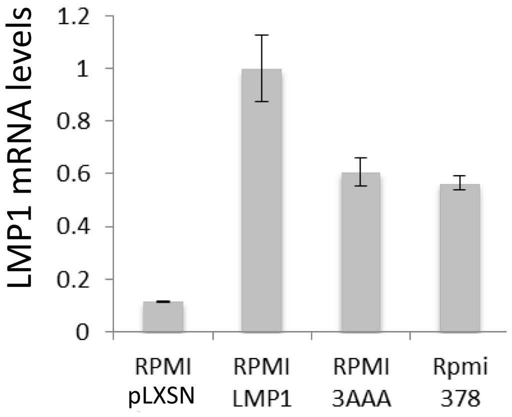

Supplementary Fig 3: **3S A** and **B)** Primary B cells from two different donors were putted in culture for 24-36h, after that cells were in part collected to make dry pellets and in part infected with WT or ∆LMP1 EBV and collected 48h post infection. WT EBV infected cells were also cultured until they got immortalized (LCL), and collected. All samples were processed for total RNA extraction. After retro-transcription samples were analyzed by qPCR for the levels of EBER (**A**) or LMP1 (**B**). Histograms show the relative mRNA average levels for the indicated genes (**p value <0.01; ns= non-significant). **3SC**) RPMI cells were stably transduced with the empty retroviral vector pLXSN (pLXSN) or with pLXSN-LMP1 (LMP1)WT or mutated (3-AAA and 378). After retro-transduction cells were cultured and collected to extract RNA. CDNA samples were interrogated by qPCR for the mRNA levels of LMP1.

Supplementary figure 3 Hernandez et al. 2017

Supplementary Table 1. The ID number, EBV status (EBV genome copy number determined by Taqman) and geographic origins of BL cell lines used in the study are indicated.

BL no	Clinical data	EBV	
		Status	EBV genome/cell
**BL101**	african	EBV+	1.802435927
**BL137**	african	EBV+	3.633117246
**BL65**	african	EBV+	1.456033409
**BL79**	african	EBV+	1.324454133
**BL110**	caucasian	EBV+	1.325302595
**BL112**	caucasian	EBV+	1.817109094
**BL116**	african	EBV+	1.249963166
**BL135**	african	EBV+	0.953680193
**BL60**	african	EBV+	1.431533383
**BL53**	caucasian	EBV-	0.000673811
**BL56**	caucasian	EBV-	0.005676779
**BL58**	caucasian	EBV-	0.00016944
**BL70**	caucasian	EBV-	0.000668915
**BL103**	caucasian	EBV-	0.004741141
**BL102**	caucasian	EBV-	0
**BL104**	caucasian	EBV-	0
**BL2**	caucasian	EBV-	0.018021102
**BL41**	caucasian	EBV-	0.014766113

Supplementary Table 2. Genes differentially expressed in 5 EBV (+) vs. 5 EBV (-) BL.

Probe_Id	Array_Address_Id	Symbol	logFC	AveExpr	t	P.Value	B
ILMN_1678904	1240300	ENO3	-1.7724	8.1166	-8.15189	1.81E-05	-1.96491
ILMN_2043452	5720192	FANCE	-1.04782	8.766813	-7.54028	3.39E-05	-2.04523
ILMN_2349658	4220091	TSPO	3.065823	6.587346	6.817012	7.46E-05	-2.16112
ILMN_1652163	7570059	DVL2	-1.03233	7.198815	-6.63196	9.22E-05	-2.19512
ILMN_1672925	510050	LOC652837	1.683532	5.590941	6.07127	0.00018	-2.31106
ILMN_1795865	4480220	FGFRL1	-1.92576	6.56506	-6.03477	0.000188	-2.31935
ILMN_1698478	1690711	SNAPC2	-1.6986	7.310377	-6.00528	0.000195	-2.32611
ILMN_1682226	3780291	CLDN15	-1.15652	8.670578	-5.91006	0.000219	-2.3484
ILMN_3179620	4040343	LOC100129673	-2.01304	9.010909	-5.76668	0.000262	-2.38327
ILMN_1805863	5130594	WDR81	-1.30952	6.55338	-5.69548	0.000287	-2.4012
ILMN_1729217	3940564	FAM131A	1.711864	5.726268	5.676255	0.000294	-2.4061
ILMN_1775573	2190544	DPY19L4	1.613885	5.611729	5.66291	0.0003	-2.40953
ILMN_2264011	5270544	GRAP	-1.60567	10.73337	-5.48678	0.000376	-2.45617
ILMN_3238435	6510156	SNORA12	2.226275	9.613034	5.474137	0.000382	-2.45962
ILMN_1807719	5910541	CTNS	-1.49004	7.61242	-5.43555	0.000402	-2.47024
ILMN_1792910	6760255	MNT	-1.18354	8.023757	-5.30256	0.000479	-2.50788
ILMN_1676191	5910097	DARS2	-1.40919	8.848535	-5.22384	0.000532	-2.53094
ILMN_1696187	2760427	PYGL	-1.64624	6.92307	-5.09147	0.000637	-2.57106
ILMN_3179309	5670689	LOC100129878	-1.00427	5.608932	-5.0718	0.000654	-2.57717
ILMN_1808196	6770730	GSTO1	2.405072	9.798916	5.037707	0.000685	-2.58785
ILMN_2205032	2760689	MAGEE1	-1.99656	6.327112	-5.02833	0.000694	-2.59081
ILMN_1672135	1570338	ZNF615	1.793808	6.314548	4.928882	0.000796	-2.62275
ILMN_3245564	840010	RICH2	-2.64381	7.410673	-4.88191	0.000849	-2.6382
ILMN_1781010	6250435	ARHGEF3	-2.95179	8.176318	-4.83899	0.000901	-2.65252
ILMN_2332964	1300452	LGMN	1.77001	7.025627	4.793195	0.000961	-2.66803
ILMN_1723139	4390332	GPD2	1.003221	5.053692	4.791776	0.000963	-2.66851
ILMN_1658995	7160592	ACOT9	2.930888	6.398779	4.779541	0.00098	-2.67269
ILMN_1724708	6270301	RAB33A	3.071323	6.207975	4.746804	0.001026	-2.68396
ILMN_1694569	6550441	LOC645100	1.163032	5.731871	4.696381	0.001102	-2.70156
ILMN_2065022	110575	KIAA0672	-2.64567	7.186033	-4.63491	0.001202	-2.7234
ILMN_1815158	4900170	GPS2	-1.36648	7.676157	-4.61506	0.001237	-2.73054
ILMN_1685005	4670750	TNFRSF1A	2.340403	6.200605	4.605126	0.001255	-2.73413
ILMN_1810891	5870687	ZNF629	1.5296	5.79278	4.59529	0.001272	-2.7377
ILMN_2381296	3450273	GSTZ1	-1.25179	8.058575	-4.58498	0.001291	-2.74145
ILMN_1779171	1940273	SGSM2	-1.43146	10.01167	-4.52525	0.001407	-2.76343
ILMN_1759870	6200113	LOC653066	-1.19301	6.177307	-4.51986	0.001418	-2.76544
ILMN_3182422	4810014	LOC100129828	-1.95485	7.993003	-4.51069	0.001437	-2.76886
ILMN_1689652	5560112	RNMTL1	-1.19132	9.470618	-4.50891	0.001441	-2.76952
ILMN_1778203	1740040	CLN5	1.436745	5.92715	4.474756	0.001514	-2.78235
ILMN_1810423	160204	RPP40	-1.37458	9.860546	-4.47145	0.001521	-2.78359
ILMN_1764871	5810630	PIGP	1.409707	9.124012	4.434393	0.001605	-2.79768
ILMN_1727390	3780239	ATPAF2	-1.53768	6.563541	-4.41169	0.001659	-2.80639
ILMN_1813795	1850592	RASSF5	1.039185	5.122927	4.363255	0.001781	-2.82519
ILMN_1694514	1450400	ZDHHC11	-2.18162	6.873242	-4.35117	0.001813	-2.82993
ILMN_2413318	5570575	C15orf57	1.463532	6.729101	4.348659	0.001819	-2.83091
ILMN_1678805	6290292	POMT2	-1.27461	6.926359	-4.3452	0.001828	-2.83227
ILMN_1675483	2760102	ANKMY1	-1.81843	7.800257	-4.33549	0.001855	-2.8361
ILMN_2183687	6420450	LIME1	-1.07286	9.935499	-4.32917	0.001872	-2.83859

ILMN_3224934	1580204	SFRS18	1.070467	10.03468	4.325942	0.001881	-2.83987
ILMN_1821280	510332		-1.62146	7.970817	-4.29308	0.001974	-2.85294
ILMN_1653026	4830113	PLAC8	3.537063	9.162536	4.220041	0.0022	-2.88247
ILMN_1761262	4010041	MPI	1.243056	6.284408	4.218392	0.002205	-2.88314
ILMN_2093343	2490372	PLAC8	4.114122	9.34543	4.214256	0.002219	-2.88483
ILMN_1690907	5890470	CCR6	2.664397	6.696884	4.213421	0.002221	-2.88518
ILMN_2168314	6420739	PGM3	1.49931	6.26854	4.203179	0.002256	-2.88938
ILMN_1694890	6350750	UCHL5IP	-1.31408	5.7872	-4.17133	0.002365	-2.90254
ILMN_1774997	4390241	BCL2L11	-1.5984	5.959666	-4.16674	0.002381	-2.90444
ILMN_2214144	940041	TWSG1	1.249954	8.91332	4.09199	0.002664	-2.93587
ILMN_2334760	2320647	ARMCX3	2.165292	6.391949	4.084084	0.002696	-2.93924
ILMN_1766499	2940577	HSPA2	1.557437	5.265807	4.0827	0.002701	-2.93983
ILMN_1669523	4280017	FOS	1.690793	5.244595	4.080228	0.002711	-2.94089
ILMN_3299558	2060433	SFRS18	1.010597	9.1495	4.075418	0.002731	-2.94294
ILMN_3226769	3290025	LOC730074	1.708661	8.29281	4.074559	0.002735	-2.94331
ILMN_2260991	1230019	TSPO	1.15783	7.40588	4.051796	0.00283	-2.95308
ILMN_1678862	70553	FUT11	1.573445	6.309429	4.050333	0.002836	-2.95371
ILMN_2044293	7210280	KBTBD7	1.53959	6.176848	4.038156	0.002889	-2.95896
ILMN_3188106	6560133	CYTH2	-1.69025	7.88263	-4.02358	0.002953	-2.96528
ILMN_1710075	6280048	FAM89A	-2.68669	7.403767	-4.02221	0.002959	-2.96587
ILMN_1740430	2070243	SLC2A4RG	-1.24572	7.55214	-4.01091	0.00301	-2.97079
ILMN_2041577	2140528	GPR172A	-1.2832	8.588126	-4.00599	0.003033	-2.97293
ILMN_1710207	6510368	C10orf6	-1.11366	8.72404	-4.00314	0.003046	-2.97418
ILMN_1712517	3310523	ZNF696	-1.12058	7.89471	-4.00143	0.003054	-2.97493
ILMN_1813019	5420528	DNAJB4	1.320402	5.459119	3.996294	0.003078	-2.97718
ILMN_1659753	3890563	LAMP2	1.796458	6.358735	3.995491	0.003082	-2.97753
ILMN_1804454	5820113	FAM135A	1.44605	6.147347	3.981007	0.00315	-2.98388
ILMN_3233739	4060373	FLJ10661	-1.00396	5.511184	-3.97314	0.003188	-2.98735
ILMN_1803018	4210561	KIFC2	-1.72818	8.014932	-3.96814	0.003212	-2.98956
ILMN_1755862	3840092	PFAS	-1.03039	10.01927	-3.96651	0.00322	-2.99028
ILMN_1762825	1070402	CANX	1.119668	8.480271	3.935917	0.003374	-3.00385
ILMN_1660749	6940484	ASPSCR1	-1.35914	10.14098	-3.90355	0.003545	-3.01835
ILMN_1693685	4180431	LOC205251	1.071226	8.15755	3.879113	0.00368	-3.02939
ILMN_1736752	6960377	COMTD1	-1.79581	8.568912	-3.87842	0.003684	-3.02971
ILMN_1682368	7100546	LRWD1	-1.26815	8.680335	-3.86597	0.003755	-3.03537
ILMN_1724250	1940021	GRN	1.490176	8.02135	3.851213	0.003841	-3.0421
ILMN_1726989	7330653	C1orf86	-1.7447	7.628796	-3.82466	0.004001	-3.05429
ILMN_1700487	7560014	TOP3A	-1.4616	6.495012	-3.81843	0.00404	-3.05716
ILMN_1734702	3060767	MANEA	1.456371	5.920329	3.817547	0.004045	-3.05757
ILMN_2383754	6760538	GTPBP10	-1.20097	6.074456	-3.81364	0.00407	-3.05937
ILMN_1781236	3170646	LOC554223	1.267468	5.136942	3.80831	0.004103	-3.06184
ILMN_1662340	6860026	ZNF358	1.721306	5.744751	3.803497	0.004134	-3.06407
ILMN_1779751	1660608	C7orf55	-1.14513	8.219633	-3.7922	0.004207	-3.06931
ILMN_2216918	5310070	SHPK	-1.27033	8.868436	-3.75759	0.004438	-3.08549
ILMN_2279961	780041	LAMP2	1.300806	9.87179	3.754277	0.004461	-3.08705
ILMN_1747281	3610148	EVI5L	-1.1346	7.239596	-3.75047	0.004487	-3.08884
ILMN_1655612	7000609	ARRDC2	-1.63932	7.507405	-3.74192	0.004547	-3.09287
ILMN_1761138	4180445	C9orf142	-1.18826	9.63473	-3.72011	0.004704	-3.1032
ILMN_1809496	2030386	COPG2	-1.52963	7.305918	-3.68285	0.004984	-3.12099
ILMN_1730816	3520598	GPR162	1.171084	5.116429	3.679711	0.005009	-3.12249
ILMN_1706959	3390373	TIMM22	-1.25424	8.541825	-3.67646	0.005034	-3.12405
ILMN_1733248	4490142	NRBP2	-1.11645	7.147616	-3.6653	0.005123	-3.12943
ILMN_1653553	7100008	C14orf80	-1.52237	8.396218	-3.66315	0.00514	-3.13047

ILMN_1774949	1820703	PIGP	2.168964	6.844328	3.661097	0.005156	-3.13146
ILMN_2415898	1710240	DNAJB14	1.095972	8.036928	3.660445	0.005162	-3.13177
ILMN_2413508	1470626	CD97	1.30478	5.546072	3.649054	0.005254	-3.13729
ILMN_1757627	4860437	ZMYND19	-1.12355	8.939183	-3.62665	0.005442	-3.14818
ILMN_1702389	4760538	ZC3H3	-1.17285	8.734467	-3.62224	0.005479	-3.15033
ILMN_1657632	7550722	ZMYM6	2.236348	8.201247	3.621824	0.005483	-3.15053
ILMN_1681679	6860753	TSPO	2.490031	9.093502	3.618132	0.005515	-3.15234
ILMN_1699987	3840181	MANEA	1.162109	5.831726	3.614316	0.005548	-3.1542
ILMN_2120072	5870072	FLJ13305	-1.32018	7.317655	-3.60934	0.005591	-3.15664
ILMN_1658677	6250092	DTX3	1.685011	5.203213	3.60362	0.005642	-3.15944
ILMN_1704369	4880537	LIMA1	1.618662	6.049577	3.601617	0.005659	-3.16043
ILMN_1723235	6270475	DUS3L	-1.23225	8.694863	-3.59819	0.00569	-3.16211
ILMN_1735038	3290332	Mar-03	1.361396	5.68713	3.594751	0.005721	-3.1638
ILMN_1674983	1940296	LOC387841	-1.39837	9.502402	-3.59381	0.005729	-3.16426
ILMN_1714158	7150167	PON2	2.05776	6.07266	3.582015	0.005836	-3.17008
ILMN_3265343	1110707	LOC100130633	2.015576	6.531509	3.578889	0.005865	-3.17162
ILMN_1714604	6110176	LOC283710	1.132057	6.421491	3.565326	0.005991	-3.17834
ILMN_1715543	4900438	ACOT1	1.8589	5.31283	3.558152	0.006059	-3.1819
ILMN_1781285	6860377	DUSP1	1.893181	6.340247	3.557002	0.00607	-3.18247
ILMN_3309739	2690630	MIRLET7D	1.058186	5.284686	3.540492	0.00623	-3.1907
ILMN_1693620	2480224	PGM3	1.665228	6.852997	3.529025	0.006344	-3.19644
ILMN_3247636	3140521	SCARNA14	2.518219	6.865165	3.525773	0.006376	-3.19807
ILMN_1656297	2900403	C21orf57	-1.59753	8.398624	-3.52542	0.00638	-3.19824
ILMN_1682312	840168	CYBB	1.513325	6.786506	3.505154	0.006587	-3.20843
ILMN_1668619	1580333	KIAA1467	1.586949	5.792122	3.504906	0.00659	-3.20856
ILMN_1798212	2470608	LLGL1	-1.22663	7.493225	-3.50332	0.006606	-3.20936
ILMN_1698311	3120274	ABHD12B	-1.4753	5.389979	-3.48714	0.006778	-3.21754
ILMN_3239445	4810181	ZBTB42	-1.53365	6.043777	-3.48523	0.006798	-3.21851
ILMN_2376529	1470414	UMODL1	-1.72768	6.557161	-3.47006	0.006963	-3.22621
ILMN_3245413	2070373	DENND5A	1.94908	7.129845	3.464502	0.007025	-3.22905
ILMN_1836309	4220184		-1.42258	6.247402	-3.45977	0.007078	-3.23146
ILMN_3300198	20450	LOC729580	1.090732	5.876948	3.457503	0.007103	-3.23262
ILMN_1802819	6200201	DEPDC1	2.009636	7.284304	3.456094	0.007119	-3.23334
ILMN_1797684	6860452	PDCD2	1.220283	9.544026	3.454497	0.007137	-3.23415
ILMN_1772976	2060364	BTNL9	-2.12963	5.393988	-3.44642	0.007229	-3.23829
ILMN_2398489	2940528	SIGMAR1	-1.36313	9.002984	-3.44235	0.007276	-3.24038
ILMN_1657797	6110008	FIBP	-1.15409	9.802981	-3.44194	0.007281	-3.24058
ILMN_1698019	4560129	LGMN	2.392158	8.09998	3.436793	0.007341	-3.24323
ILMN_2354381	3140390	PON2	2.428141	6.259623	3.436716	0.007341	-3.24327
ILMN_1660261	3440112	TDRD6	-1.46953	6.005655	-3.43271	0.007388	-3.24533
ILMN_1860288	4220059		1.240477	6.344391	3.426987	0.007456	-3.24827
ILMN_2228873	5550360	STARD3NL	1.959268	6.771715	3.425601	0.007472	-3.24898
ILMN_3237446	2030646	SCARNA16	1.511434	7.109077	3.420915	0.007528	-3.2514
ILMN_1712505	160209	KDELC1	1.088071	5.149926	3.42091	0.007528	-3.2514
ILMN_1689876	5130209	LOC648189	1.141183	5.382319	3.414688	0.007603	-3.25461
ILMN_2407811	1990292	NEURL4	-1.4652	6.270506	-3.40987	0.007661	-3.2571
ILMN_2385178	2260619	MIB2	-1.20831	7.72143	-3.40312	0.007744	-3.2606
ILMN_2229940	1170524	C2orf7	-1.21502	7.443685	-3.39457	0.00785	-3.26503
ILMN_1810531	5960017	DRG2	-1.39082	7.674128	-3.38879	0.007923	-3.26804
ILMN_1737663	4900338	C7orf13	1.240845	5.707152	3.387465	0.00794	-3.26873
ILMN_1657619	60594	DNAJB14	1.491404	6.396789	3.385558	0.007964	-3.26972
ILMN_1804117	2000224	FAM89B	-1.00036	7.964497	-3.38546	0.007965	-3.26977
ILMN_1654331	2120008	HOXB4	1.212614	5.476424	3.384653	0.007975	-3.27019

ILMN_1708907	2140626	MEIG1	-1.0337	5.333887	-3.38173	0.008012	-3.27171
ILMN_1752351	3120743	LAMP2	1.416938	9.054511	3.377474	0.008067	-3.27393
ILMN_2342793	1470592	FBXW8	-1.17783	6.404567	-3.36818	0.008187	-3.27878
ILMN_1745813	5420110	KIAA1279	1.024054	6.927651	3.367545	0.008196	-3.27912
ILMN_3268880	7330730	C10orf75	1.366467	5.20096	3.364322	0.008238	-3.2808
ILMN_2225608	6560131	FAM135A	1.452815	6.535142	3.35241	0.008396	-3.28705
ILMN_2134224	2060193	ATP13A1	-1.76981	8.541105	-3.35102	0.008415	-3.28778
ILMN_1807177	50544	KIAA1797	1.796885	6.712191	3.349062	0.008441	-3.28881
ILMN_3236656	7040349	LOC286367	-1.6626	6.472538	-3.32817	0.008728	-3.29982
ILMN_2135798	3060563	NR2C2AP	-1.10795	8.836344	-3.32338	0.008795	-3.30235
ILMN_1746561	5050156	BCL2L2	2.058896	6.479392	3.319915	0.008844	-3.30418
ILMN_1769018	1710538	PCSK4	-1.34443	5.859998	-3.31313	0.00894	-3.30778
ILMN_2072973	3890553	TOP3A	-1.91446	9.178053	-3.30846	0.009007	-3.31026
ILMN_1810922	1510014	PCNT	-1.0695	9.952572	-3.30834	0.009009	-3.31032
ILMN_1767168	5420132	CAMK4	1.111316	5.835795	3.307042	0.009028	-3.31101
ILMN_1740685	2640463	LOC652541	-1.59994	6.268946	-3.3034	0.009081	-3.31294
ILMN_1806023	6510367	JUN	1.96741	5.937417	3.302273	0.009097	-3.31354
ILMN_1724207	6020398	IVD	1.432754	6.22064	3.286676	0.009327	-3.32186
ILMN_1678919	4590020	YOD1	2.33292	7.312243	3.285548	0.009344	-3.32246
ILMN_1675117	6280008	HSD17B11	1.45726	6.191052	3.284568	0.009359	-3.32298
ILMN_1654370	460427	TESK2	1.668022	6.490357	3.281132	0.00941	-3.32482
ILMN_1794522	1850414	EIF5A	-1.18819	10.9138	-3.27481	0.009506	-3.3282
ILMN_1654778	4180068	LSP1	1.360078	7.335363	3.273191	0.009531	-3.32907
ILMN_1682332	7000577	GYPC	1.49125	9.188607	3.268248	0.009607	-3.33172
ILMN_1683082	6290181	RPUSD1	-1.16507	8.065715	-3.26671	0.009631	-3.33254
ILMN_1741942	7040315	STX16	1.174383	10.20561	3.262522	0.009696	-3.33479
ILMN_1652754	1820543	ZNF428	-1.13344	10.92617	-3.25945	0.009744	-3.33644
ILMN_1709750	7560180	SUSD1	1.168077	5.597374	3.257423	0.009776	-3.33753
ILMN_2222234	2650253	PRDX4	1.013302	10.41135	3.253757	0.009833	-3.3395
ILMN_1669033	160411	NCOA1	-1.36233	7.970432	-3.25179	0.009864	-3.34057
ILMN_1777881	3710609	TSPAN17	-1.25946	8.185098	-3.24877	0.009912	-3.34219
ILMN_1651538	4200121	NUMBL	-1.10504	5.319559	-3.24875	0.009913	-3.3422
ILMN_3246315	4290671	LOC100133697	-1.08334	7.105534	-3.24852	0.009916	-3.34232
ILMN_1777156	520035	GTPBP3	-1.42905	7.542046	-3.24804	0.009924	-3.34258
ILMN_1718044	60202	ELAC2	-1.36224	7.952299	-3.24752	0.009932	-3.34286
ILMN_1701837	1070593	KLHL2	1.969909	6.870483	3.246044	0.009956	-3.34366
ILMN_1662970	3440538	ZP3	1.051349	5.916884	3.236577	0.010109	-3.34877
ILMN_2334765	7150427	ARMCX3	1.247158	5.646557	3.230681	0.010205	-3.35196
ILMN_2400922	1440754	OPRL1	-1.33044	7.278598	-3.22859	0.010239	-3.3531
ILMN_1809437	6650746	RHBDD2	-1.38599	8.524456	-3.22491	0.0103	-3.35509
ILMN_1693334	4220731	P4HA1	1.939336	8.596474	3.213574	0.01049	-3.36124
ILMN_2336133	5490594	SULT1A4	-1.27614	7.859863	-3.21191	0.010518	-3.36215
ILMN_3226700	4780360	ZNF837	-1.41179	6.680681	-3.21071	0.010538	-3.3628
ILMN_2312340	540671	LILRB2	1.594301	5.417556	3.210257	0.010546	-3.36305
ILMN_1746243	2120402	TES	-1.82176	6.139385	-3.20861	0.010574	-3.36394
ILMN_1770719	7400711	KIAA0664	-1.35346	8.337673	-3.19979	0.010725	-3.36875
ILMN_1794782	6060377	ABCG1	1.776031	6.098654	3.192315	0.010855	-3.37283
ILMN_2077680	610154	CLDND2	-1.47371	6.854952	-3.18596	0.010967	-3.37631
ILMN_1735955	3450722	LOC644033	-1.36312	9.325409	-3.18151	0.011046	-3.37874
ILMN_2073235	4730181	FTHL12	1.990904	10.28444	3.1794	0.011084	-3.3799
ILMN_2141482	6290356	SERPINF1	-1.38254	5.289062	-3.17777	0.011113	-3.38079
ILMN_1874678	2640577		-1.07331	5.538846	-3.17712	0.011125	-3.38115
ILMN_1680727	6980274	GLRX2	-1.02402	8.760281	-3.17704	0.011126	-3.38119

ILMN_1792682	990349	MCTP2	1.108306	5.516589	3.173322	0.011193	-3.38323
ILMN_1767459	4780253	POLR3B	-1.38581	8.41272	-3.17046	0.011245	-3.38481
ILMN_1768534	2640735	BHLHB2	2.575566	7.67162	3.167024	0.011308	-3.38669
ILMN_1745806	1780411	PEMT	-1.44724	8.242505	-3.16677	0.011312	-3.38683
ILMN_3269225	6840070	LOC100128895	-1.00641	5.408484	-3.16323	0.011377	-3.38878
ILMN_2326591	450671	ANXA6	1.34538	5.239065	3.159304	0.011449	-3.39094
ILMN_1661636	5270088	ZMYM2	1.037605	7.946613	3.144553	0.011726	-3.39908
ILMN_1798108	2750129	C6orf211	1.497188	6.499145	3.138219	0.011846	-3.40258
ILMN_2384807	1500681	LRRCC1	-1.46148	9.31095	-3.13287	0.011949	-3.40555
ILMN_1724293	1500102	KDELR2	1.212179	8.836915	3.132371	0.011959	-3.40582
ILMN_3238854	670301	RGPD8	1.156776	5.792977	3.132214	0.011962	-3.40591
ILMN_1735367	6580487	HSD17B11	2.126288	7.416882	3.119309	0.012214	-3.41308
ILMN_2203950	3400438	HLA-A	1.405749	12.4457	3.118938	0.012222	-3.41328
ILMN_1705111	7200358	FNDC3A	1.972274	6.917681	3.117367	0.012253	-3.41416
ILMN_1694084	6860719	PSCD1	1.171342	10.35718	3.115926	0.012282	-3.41496
ILMN_1747506	3310725	DHX34	-1.12842	7.599282	-3.1156	0.012288	-3.41514
ILMN_1668752	6130164	FLJ20850	-1.47142	6.345189	-3.11123	0.012375	-3.41757
ILMN_1806713	1230274	ZNF18	-1.29652	7.286521	-3.11062	0.012387	-3.41791
ILMN_1654629	1090564	TMEM175	-1.34761	8.150114	-3.10964	0.012407	-3.41846
ILMN_2310589	4890717	DIABLO	-1.23309	11.26832	-3.1094	0.012412	-3.41859
ILMN_1657409	5720379	SERHL	-1.78182	5.77742	-3.10751	0.01245	-3.41964
ILMN_1806752	1780019	PLEKHH2	1.011254	5.042442	3.103229	0.012537	-3.42203
ILMN_1677785	2940647	ZNF559	-2.02131	7.802641	-3.10062	0.01259	-3.42349
ILMN_2230566	510484	RAB40B	-1.19101	8.215015	-3.0925	0.012757	-3.42803
ILMN_2048388	5860367	ZNF643	-1.45909	6.642634	-3.09246	0.012757	-3.42805
ILMN_1687303	6560327	ACAD10	-1.25746	7.536727	-3.09189	0.012769	-3.42837
ILMN_1724734	1170095	UQCC	1.109199	5.600694	3.09122	0.012783	-3.42874
ILMN_1709814	540524	NMRAL1	-1.40751	10.12773	-3.09074	0.012793	-3.42901
ILMN_3242234	7560292	LOC100134018	-1.41111	5.322828	-3.07003	0.01323	-3.44063
ILMN_1681437	6660132	DCXR	-1.10822	9.654512	-3.06987	0.013234	-3.44072
ILMN_1734895	5360521	SFT2D1	2.495926	10.23398	3.065278	0.013333	-3.4433
ILMN_1665243	6100411	FKBP14	1.100324	6.655759	3.059425	0.01346	-3.4466
ILMN_3239548	3060601	LOC100132740	-1.16857	8.75683	-3.05881	0.013473	-3.44695
ILMN_3240222	2640441	PRAGMIN	-1.29133	9.826934	-3.05648	0.013524	-3.44826
ILMN_3233135	7330563	FAM178A	-1.14276	7.519174	-3.05408	0.013577	-3.44962
ILMN_1773780	1470561	FAM173A	-1.02666	9.145182	-3.05354	0.013589	-3.44992
ILMN_2101832	580132	LAPTM4B	3.113312	6.369856	3.052487	0.013612	-3.45052
ILMN_2318568	50088	HCFC1R1	-1.31431	7.622179	-3.05102	0.013645	-3.45135
ILMN_2367707	2360474	PKN1	-1.37972	8.525675	-3.04092	0.01387	-3.45705
ILMN_1803110	1090239	SF3B3	-1.43643	9.113227	-3.04041	0.013882	-3.45734
ILMN_1709333	1570156	OAS2	1.287342	5.659671	3.03397	0.014028	-3.46099
ILMN_1780711	3800202	LOC390530	1.408866	6.113829	3.029517	0.01413	-3.46352
ILMN_1855430	5960735	LOC199800	-1.371	6.762483	-3.0154	0.014458	-3.47154
ILMN_1707051	940725	NFATC1	-1.2908	8.37516	-3.01255	0.014526	-3.47317
ILMN_1813191	4060685	LOC653080	-1.36256	8.03602	-3.00977	0.014591	-3.47475
ILMN_1676588	2190730	CEPT1	1.137462	10.01701	3.009512	0.014597	-3.47489
ILMN_1813685	4540370	RAB7L1	1.897406	6.463394	3.007313	0.01465	-3.47615
ILMN_1731107	4830682	CCDC92	-1.03466	7.204218	-3.00703	0.014657	-3.47631
ILMN_2134381	360373	C14orf85	-1.07624	12.53773	-3.00634	0.014673	-3.4767
ILMN_2285817	7400747	FAM89A	-2.00251	6.94632	-3.00469	0.014712	-3.47764
ILMN_2252408	6200743	CNPY4	-1.08563	7.847458	-3.00265	0.014761	-3.47881
ILMN_1668039	7210398	GYPC	1.244214	9.961175	3.000279	0.014819	-3.48016
ILMN_2352216	1580215	FBXL6	-1.11363	7.643763	-2.99726	0.014891	-3.48188

ILMN_1753950	3830370	ZNF326	-1.16857	6.561654	-2.99714	0.014894	-3.48195
ILMN_2329927	5860377	ABCG1	1.405516	5.124987	2.996562	0.014908	-3.48229
ILMN_2302118	70603	CCDC50	1.786296	9.685619	2.994333	0.014963	-3.48356
ILMN_1673282	3060292	LAMP2	1.868445	7.021765	2.993944	0.014972	-3.48378
ILMN_1791375	3710711	STAG3L2	-1.26699	10.05589	-2.99295	0.014996	-3.48435
ILMN_2103480	2690711	ZNF320	-1.33401	8.146994	-2.99133	0.015036	-3.48528
ILMN_1756439	1030239	SCRN1	2.547246	7.734073	2.988961	0.015094	-3.48663
ILMN_3245057	6840129	ASAP1	-1.02203	9.430356	-2.98836	0.015109	-3.48698
ILMN_1780153	2100563	QTRT1	-1.60268	7.145649	-2.98515	0.015188	-3.48882
ILMN_2406410	510373	RHBDD2	-1.2554	8.294341	-2.9837	0.015224	-3.48965
ILMN_1748831	3420228	PPP1R13B	-1.87142	7.256054	-2.98322	0.015236	-3.48992
ILMN_1726516	4290196	SCRIB	-1.07042	9.305672	-2.98291	0.015244	-3.4901
ILMN_1734229	4920398	SPPL2A	1.477268	7.950677	2.981335	0.015283	-3.491
ILMN_1772296	4210286	C17orf68	-1.31546	6.36234	-2.98132	0.015283	-3.49101
ILMN_2148668	1400035	RCBTB2	1.750871	6.293821	2.978656	0.01535	-3.49254
ILMN_2410986	2100484	STAT3	1.372794	5.779407	2.977831	0.01537	-3.49301
ILMN_2362581	1660113	FNDC3A	1.887514	6.901513	2.976735	0.015398	-3.49364
ILMN_1654112	460086	PARD6A	-1.14993	7.912108	-2.97066	0.015551	-3.49713
ILMN_1812940	7650278	TRMT1	-1.32372	9.479953	-2.96812	0.015616	-3.49859
ILMN_1683120	3610750	UNG	-1.40342	9.872601	-2.9655	0.015682	-3.5001
ILMN_1773764	1780274	CECR7	-1.6637	7.121839	-2.96521	0.01569	-3.50026
ILMN_1701613	2650564	RARRES3	1.625749	6.676207	2.963503	0.015734	-3.50125
ILMN_1775566	5570152	ATP1A1	2.193286	7.884483	2.960685	0.015806	-3.50287
ILMN_1687978	5900725	PHLDA1	1.433197	5.509154	2.956905	0.015904	-3.50505
ILMN_1748093	1510521	PAFAH1B3	-1.17173	10.28281	-2.95537	0.015944	-3.50593
ILMN_1717925	510136	SIGMAR1	-1.04167	6.358922	-2.95524	0.015947	-3.50601
ILMN_3288717	2750685	LOC392437	1.573318	9.801927	2.953767	0.015985	-3.50686
ILMN_2113738	4640132	C8orf45	-1.14313	12.42025	-2.952	0.016032	-3.50788
ILMN_1774513	5130692	DDX17	1.387228	6.368214	2.95065	0.016067	-3.50866
ILMN_2090802	520195	TMEM79	-1.51863	7.689583	-2.95004	0.016083	-3.50901
ILMN_1716237	6290059	ACOT2	1.461535	5.434841	2.949625	0.016094	-3.50925
ILMN_2231298	5260064	SERHL2	-1.29017	5.264832	-2.94809	0.016134	-3.51014
ILMN_2374383	840403	TSPAN17	-1.00544	6.137739	-2.94599	0.01619	-3.51135
ILMN_1789500	1510731	KIAA1875	-1.22193	6.211778	-2.94584	0.016194	-3.51143
ILMN_2214098	4220270	BIVM	2.34126	6.257386	2.944803	0.016221	-3.51203
ILMN_1691567	7550639	GNPDA2	1.631337	7.441554	2.943524	0.016255	-3.51277
ILMN_2401978	5090619	STAT3	1.779452	7.423745	2.941017	0.016322	-3.51422
ILMN_1804663	1260039	THBS3	-1.42641	6.623998	-2.94093	0.016324	-3.51427
ILMN_1753241	5690431	SNTA1	-1.19711	7.330701	-2.93935	0.016366	-3.51519
ILMN_1676629	2070039	INSIG2	1.943952	7.344806	2.936026	0.016455	-3.51711
ILMN_1674201	6860592	LOC441511	1.654828	6.433335	2.935111	0.01648	-3.51764
ILMN_3230337	6580500	LOC100132707	-1.86304	6.67805	-2.93441	0.016499	-3.51805
ILMN_1684964	5560411	ZNF212	-1.29858	8.189632	-2.93255	0.016549	-3.51912
ILMN_3304405	4610022	NCRNA00081	1.286998	6.423468	2.931736	0.016571	-3.5196
ILMN_1814823	5810328	FTL	1.56338	12.37868	2.931607	0.016574	-3.51967
ILMN_1670134	2360020	FADS1	2.595186	5.97351	2.930723	0.016598	-3.52018
ILMN_1655244	5860242	LOC642755	1.064271	6.742061	2.930133	0.016614	-3.52052
ILMN_1761309	2480709	ADCK5	-1.40473	6.684342	-2.92755	0.016684	-3.52202
ILMN_2312194	5810414	CYB5A	2.686233	7.751291	2.927291	0.016691	-3.52217
ILMN_1718353	3830520	LOC652458	1.054132	5.875416	2.926973	0.0167	-3.52236
ILMN_2064606	2850039	TBC1D2B	1.659527	8.12478	2.926091	0.016724	-3.52287
ILMN_1662896	2140139	BRWD2	1.267647	7.446146	2.92601	0.016726	-3.52292
ILMN_1708267	7100328	CLDN15	-1.26652	5.650337	-2.92595	0.016728	-3.52295

ILMN_3239856	7160730	NCRNA00120	1.048403	5.522482	2.923526	0.016794	-3.52436
ILMN_2143148	10386	TM2D1	1.116429	9.836062	2.922503	0.016822	-3.52495
ILMN_1714438	5670037	MUTYH	-1.01706	9.096155	-2.92147	0.016851	-3.52555
ILMN_1808919	6280189	HPS4	-1.27534	7.833679	-2.92073	0.016871	-3.52598
ILMN_1800540	830274	CD55	1.295088	6.416926	2.919547	0.016904	-3.52667
ILMN_3236010	5310066	SCARNA11	1.401134	5.539961	2.915369	0.01702	-3.5291
ILMN_2376416	6020246	UBXN11	-1.4591	7.554103	-2.91213	0.01711	-3.53098
ILMN_2126423	3520537	ZNF480	-2.00271	8.453478	-2.90866	0.017207	-3.533
ILMN_3227315	2810463	LOC729009	1.30404	9.057068	2.907968	0.017227	-3.5334
ILMN_1660965	2970746	PRR22	-2.34957	6.335745	-2.90677	0.01726	-3.5341
ILMN_1688464	2680538	MAP6D1	-1.02087	6.502857	-2.90104	0.017423	-3.53744
ILMN_1682495	2760224	FOXP1	-1.31428	6.738728	-2.90025	0.017445	-3.5379
ILMN_1665686	2510753	FAM38B	-1.83624	6.29561	-2.89855	0.017494	-3.53889
ILMN_1840316	6100446		1.085705	5.281286	2.897491	0.017524	-3.53951
ILMN_1810782	3450059	SH3KBP1	1.538748	10.66685	2.897356	0.017528	-3.53959
ILMN_1807535	510619	YWHAE	-1.17862	7.777443	-2.89588	0.017571	-3.54045
ILMN_1811636	5700224	IFT57	-1.18868	6.926444	-2.89496	0.017597	-3.54099
ILMN_1794349	2060044	XYLB	-2.01995	6.088935	-2.89383	0.017629	-3.54165
ILMN_1706434	5870307	LOC440359	1.792429	8.66716	2.893262	0.017646	-3.54198
ILMN_3251155	3310427	PCBP2	-1.10392	7.521251	-2.89269	0.017662	-3.54231
ILMN_1677793	2900427	P2RX5	1.907324	6.424597	2.892675	0.017663	-3.54232
ILMN_1789349	3610487	UBQLN4	-1.49689	10.17397	-2.88981	0.017746	-3.544
ILMN_1803988	610750	MCL1	1.285298	9.696486	2.886016	0.017856	-3.54621
ILMN_1775708	3800168	SLC2A3	2.584616	8.504194	2.881521	0.017988	-3.54885
ILMN_2078697	540390	ALPK1	1.071558	7.592359	2.875576	0.018164	-3.55233
ILMN_1667432	2680202	HYAL3	-1.49874	5.96769	-2.87546	0.018167	-3.55239
ILMN_1689389	7330068	SF3B5	1.232332	9.958528	2.874794	0.018187	-3.55279
ILMN_1747744	360132	LHFPL2	1.566488	7.570434	2.874171	0.018206	-3.55315
ILMN_1720373	270152	SLC7A5	-1.26543	10.90982	-2.87359	0.018223	-3.55349
ILMN_1656537	2480291	SNRPN	2.936105	8.156711	2.871464	0.018286	-3.55474
ILMN_1751081	3870561	EHMT2	-1.13388	6.91324	-2.86608	0.018448	-3.5579
ILMN_1811836	5720609	DYNC2LI1	1.931188	7.154158	2.86441	0.018499	-3.55888
ILMN_1758337	5130332	ZNF213	-1.14046	6.743007	-2.85887	0.018667	-3.56214
ILMN_1698185	5700431	WDR90	-1.09086	8.287151	-2.85692	0.018727	-3.56328
ILMN_1663667	4290014	FLAD1	-1.17203	9.222508	-2.85268	0.018857	-3.56578
ILMN_1788131	5270072	CYP26A1	-3.14576	6.075247	-2.84835	0.018992	-3.56833
ILMN_2384513	3460605	C2CD2	1.044809	5.408552	2.843914	0.01913	-3.57094
ILMN_1783337	380148	DECR2	-1.58825	7.142373	-2.84386	0.019132	-3.57098
ILMN_1688178	3130598	RRP7A	-1.15431	10.81013	-2.83924	0.019277	-3.5737
ILMN_3240538	870133	C19orf38	-1.11207	5.842643	-2.83889	0.019288	-3.57391
ILMN_3200854	4040110	LOC441453	-1.59615	5.494426	-2.83639	0.019367	-3.57538
ILMN_1735157	4880544	GALNT12	-1.19631	6.528592	-2.83188	0.019511	-3.57805
ILMN_1794823	630767	ZNF626	1.389072	6.290189	2.826012	0.019699	-3.58152
ILMN_1757026	4610091	MANEA	1.653612	6.796816	2.825232	0.019725	-3.58198
ILMN_1738530	4150086	ATAD3A	-1.23381	9.001217	-2.82131	0.019852	-3.5843
ILMN_1660000	1050408	SNURF	1.98861	8.890831	2.815411	0.020045	-3.5878
ILMN_1849013	4010100		1.755367	6.377253	2.815304	0.020048	-3.58787
ILMN_1676905	4640438	TIGD7	1.437243	6.222478	2.813242	0.020116	-3.58909
ILMN_1684553	160674	RHOH	1.54079	7.411645	2.811166	0.020185	-3.59032
ILMN_1665717	2850440	EIF2S3	1.212117	11.29022	2.810883	0.020194	-3.59049
ILMN_2401878	4830315	DUSP10	1.592716	6.771745	2.810698	0.0202	-3.5906
ILMN_1667418	5290136	LOC283953	1.101879	5.74639	2.810173	0.020217	-3.59091
ILMN_2235086	4920082	CCDC4	1.216728	5.788394	2.808332	0.020279	-3.592

ILMN_3231944	6380037	LOC100130516	-1.49178	10.98493	-2.80819	0.020283	-3.59209
ILMN_3209358	3310521	LOC646836	1.370839	6.125622	2.80616	0.020351	-3.59329
ILMN_1755974	7330544	ALDOC	1.520337	9.997661	2.805654	0.020368	-3.59359
ILMN_1685679	1090594	RNF146	1.053128	5.999453	2.804497	0.020406	-3.59428
ILMN_1708721	360221	RABGAP1L	-1.26754	9.093763	-2.80223	0.020482	-3.59563
ILMN_1664815	4390064	ELK4	-1.666	5.314456	-2.80182	0.020496	-3.59587
ILMN_1764383	6450056	MCOLN1	-1.484	5.86841	-2.7957	0.020703	-3.59951
ILMN_3237270	780672	LOC100133609	1.491853	6.099734	2.795078	0.020724	-3.59988
ILMN_1787256	6480674	HCN3	-1.38347	6.471142	-2.79113	0.020859	-3.60224
ILMN_1714700	2570328	TRIB2	-2.09906	7.525197	-2.78894	0.020934	-3.60354
ILMN_2371700	1090370	UCHL5IP	-1.06394	9.51669	-2.78761	0.020979	-3.60433
ILMN_1667430	5560398	DEGS1	1.354533	7.981504	2.784957	0.021071	-3.60591
ILMN_1696911	1980594	FTHL8	1.494166	9.977993	2.782332	0.021162	-3.60748
ILMN_2405756	2350541	VAMP1	1.001253	5.6012	2.780697	0.021219	-3.60846
ILMN_1711199	510767	ZNF331	-1.27999	7.300087	-2.77954	0.021259	-3.60914
ILMN_1704261	6370719	RANGRF	-1.45868	6.153375	-2.77948	0.021261	-3.60918
ILMN_2051900	2350543	EID2B	-1.22201	12.10149	-2.77409	0.02145	-3.6124
ILMN_1675577	4150484	TRMT61A	-1.53957	7.512159	-2.77199	0.021524	-3.61365
ILMN_1656900	6270725	SULT1A1	-1.10731	7.920132	-2.7695	0.021612	-3.61514
ILMN_1702044	4560482	CNTROB	-1.47736	6.337691	-2.76816	0.02166	-3.61595
ILMN_2183389	3400349	TTC9C	1.148681	7.551856	2.765413	0.021758	-3.61759
ILMN_1811171	5870692	GPR132	1.612269	5.420695	2.762696	0.021855	-3.61922
ILMN_1708248	20246	LILRB1	1.495286	5.293143	2.762464	0.021863	-3.61936
ILMN_2228453	6650288	KIAA0562	-1.08656	7.119974	-2.76161	0.021894	-3.61987
ILMN_1731287	5090056	ARFGAP3	2.320101	7.641633	2.756179	0.02209	-3.62312
ILMN_3264466	5360735	FAM54B	1.076749	5.993778	2.755861	0.022102	-3.62331
ILMN_1798706	4490520	EBI2	2.987149	6.898391	2.755626	0.02211	-3.62345
ILMN_2367753	6900630	ATP2B4	1.237229	5.865759	2.754506	0.022151	-3.62413
ILMN_2347949	4200450	G6PD	-1.31652	8.978632	-2.75428	0.022159	-3.62426
ILMN_1707925	2690040	ABHD12B	-1.93169	5.614522	-2.75088	0.022283	-3.6263
ILMN_1799208	1980092	CSGALNACT2	1.070987	5.700822	2.750227	0.022307	-3.62669
ILMN_2130441	5870521	HLA-H	2.30672	11.0608	2.749035	0.022351	-3.62741
ILMN_1660436	3850433	HSPA1B	1.665295	7.045169	2.747718	0.022399	-3.6282
ILMN_1767365	6250685	PAK1	1.757628	6.986441	2.747519	0.022406	-3.62832
ILMN_3247261	4120615	RAPGEF2	2.666972	7.165501	2.744969	0.0225	-3.62985
ILMN_1711792	6480315	GPBP1	1.058106	9.577294	2.74333	0.022561	-3.63084
ILMN_1683658	5050427	FKBP1A	1.02492	10.33669	2.743227	0.022565	-3.6309
ILMN_1743187	5560703	C6orf120	1.047027	5.802462	2.740164	0.022679	-3.63274
ILMN_1750278	3190133	FTHL12	1.074776	10.24127	2.738466	0.022742	-3.63376
ILMN_1673363	6960630	CD97	2.432377	8.49038	2.738068	0.022757	-3.634
ILMN_1677098	1110465	YPEL2	1.532268	5.968162	2.737016	0.022796	-3.63463
ILMN_1782788	2320129	CSDA	2.189577	11.25766	2.736747	0.022806	-3.63479
ILMN_1757660	6760450	CAPS	-1.08409	6.777427	-2.73625	0.022825	-3.63509
ILMN_1741613	2680487	SERINC1	1.36697	6.919537	2.734491	0.022891	-3.63615
ILMN_1683146	5220240	FTH1	1.266191	8.159629	2.734058	0.022907	-3.63641
ILMN_2081335	3930653	C7orf44	1.346013	10.36598	2.732784	0.022955	-3.63718
ILMN_2207363	70634	RABAC1	-1.02986	9.093014	-2.73143	0.023006	-3.63799
ILMN_3181480	3290537	FLJ36131	-1.59469	11.15593	-2.73088	0.023027	-3.63833
ILMN_1761031	3520220	PTPDC1	1.331719	5.40775	2.730427	0.023044	-3.6386
ILMN_1693401	7330575	KLHL28	1.111172	6.970776	2.730304	0.023049	-3.63867
ILMN_1760347	7550484	SRGN	2.593523	9.568036	2.726998	0.023175	-3.64066
ILMN_1745242	3890609	PLSCR1	1.182065	5.203308	2.726776	0.023183	-3.6408
ILMN_1680419	2190528	ASB7	-1.1972	6.926182	-2.72567	0.023225	-3.64146

ILMN_1683598	7000307	ACSL4	2.343951	8.147194	2.725322	0.023238	-3.64167
ILMN_1813657	4210241	PHF20	1.14082	6.956679	2.722433	0.023349	-3.64342
ILMN_2289844	70750	SERHL	-1.36167	6.625927	-2.72232	0.023353	-3.64349
ILMN_1720889	5860348	SC4MOL	1.429722	9.007323	2.721466	0.023386	-3.644
ILMN_1715583	1660435	BOP1	-1.05516	11.15478	-2.71976	0.023452	-3.64503
ILMN_1769822	4890470	PLAA	1.053282	5.147582	2.719313	0.023469	-3.6453
ILMN_1761464	4560047	CD74	1.948947	6.798529	2.716938	0.023561	-3.64673
ILMN_1785356	2640408	DENND5A	1.998003	7.901169	2.716883	0.023563	-3.64677
ILMN_2126832	4880463	SEC24A	1.355574	6.841848	2.715445	0.023619	-3.64763
ILMN_1799744	3440491	GALC	1.879567	6.87896	2.713233	0.023705	-3.64897
ILMN_1664283	1440050	C9orf75	-1.00904	6.855315	-2.71111	0.023787	-3.65025
ILMN_1653896	7210438	ATRIP	-1.48776	7.890199	-2.71019	0.023823	-3.65081
ILMN_1761566	4540241	C5orf32	1.654578	7.290704	2.709734	0.023841	-3.65108
ILMN_3236346	3780079	LOC100132901	1.525417	6.181642	2.709228	0.023861	-3.65139
ILMN_2169152	360500	SRGN	2.559604	9.718415	2.708678	0.023883	-3.65172
ILMN_2401873	3780707	DUSP10	1.18853	5.981315	2.707746	0.023919	-3.65229
ILMN_1715885	3400440	PTPN22	1.351171	7.2089	2.706118	0.023983	-3.65327
ILMN_2234016	2970431	FTHL7	2.146815	11.62567	2.70494	0.02403	-3.65398
ILMN_1747924	6760747	LOC644254	1.319	6.472021	2.704343	0.024054	-3.65434
ILMN_1695847	6330102	ZKSCAN5	-1.13951	6.43896	-2.70176	0.024156	-3.65591
ILMN_1731085	1690368	LOC440864	-2.47633	9.576743	-2.70092	0.024189	-3.65641
ILMN_1764609	2650020	PWWP2B	1.466932	6.211647	2.700582	0.024203	-3.65662
ILMN_1668374	2650114	ITGB5	1.398358	5.407514	2.699173	0.024259	-3.65747
ILMN_1725889	3890088	AFF2	-1.96074	7.206019	-2.69808	0.024302	-3.65814
ILMN_1728684	3310041	PELP1	-1.51378	9.312536	-2.69672	0.024357	-3.65896
ILMN_1746309	2940192	LOC389791	-1.05231	6.6404	-2.69652	0.024365	-3.65908
ILMN_1757877	5420347	HCFC1R1	-1.02225	9.713635	-2.69476	0.024435	-3.66014
ILMN_1698770	2760349	C5orf33	-1.00146	7.029192	-2.69465	0.02444	-3.66021
ILMN_1703301	1710280	LOC653479	1.155275	8.783827	2.69161	0.024562	-3.66206
ILMN_1743103	3460451	SH3PXD2A	2.117939	6.371171	2.691553	0.024565	-3.66209
ILMN_1672717	1170398	C10orf57	1.361065	6.664792	2.690468	0.024608	-3.66275
ILMN_2321578	1980184	P2RY10	2.107885	7.3968	2.68905	0.024666	-3.66361
ILMN_1783798	6860291	GAS8	1.002697	5.108124	2.688884	0.024673	-3.66371
ILMN_2164164	6110358	AICDA	2.047371	7.151045	2.688115	0.024704	-3.66418
ILMN_1810719	5220670	DCUN1D1	1.352177	6.967635	2.687865	0.024714	-3.66433
ILMN_1802627	2650138	PSMG3	-1.07358	7.847259	-2.68583	0.024797	-3.66556
ILMN_1703041	7400259	IDUA	-1.21011	5.196248	-2.68533	0.024817	-3.66587
ILMN_1700762	2760544	PBX4	-1.33964	6.608023	-2.68516	0.024824	-3.66597
ILMN_1746206	3390605	AZI1	-1.44473	8.655336	-2.68513	0.024826	-3.66599
ILMN_1799853	7570370	SPTLC1	1.254636	7.129395	2.68467	0.024844	-3.66627
ILMN_1766182	4640609	GNAZ	-1.5011	6.257639	-2.68402	0.024871	-3.66666
ILMN_3226276	3120494	LOC729799	-1.08778	5.775542	-2.68215	0.024947	-3.66779
ILMN_2330584	4560242	DDX19B	1.213219	7.224875	2.682028	0.024952	-3.66787
ILMN_2232430	4010048	NMD3	1.227434	9.133493	2.679396	0.025061	-3.66947
ILMN_2232478	4120369	APOBEC3G	1.862231	7.871254	2.67835	0.025104	-3.67011
ILMN_1765446	3800452	EMP3	1.437728	8.820363	2.673388	0.025309	-3.67312
ILMN_1680196	6650348	LAPTM4B	2.428825	6.965371	2.671814	0.025375	-3.67408
ILMN_1813833	2970475	NARFL	-1.16377	7.67181	-2.67173	0.025378	-3.67413
ILMN_1669842	2120097	CHAF1A	-1.5229	7.761576	-2.66962	0.025467	-3.67542
ILMN_1689007	5090253	SFRS14	-1.04263	7.8247	-2.66927	0.025481	-3.67563
ILMN_3213185	1230370	LOC645452	-1.20831	12.28808	-2.66821	0.025526	-3.67627
ILMN_1718808	460020	AKAP10	1.211747	6.354968	2.667744	0.025545	-3.67656
ILMN_1766762	6580369	DYNLRB1	1.151291	8.551181	2.667389	0.02556	-3.67677

ILMN_3300358	3290687	ZNF84	-1.06902	7.884769	-2.66707	0.025573	-3.67696
ILMN_1793201	6900431	HAGHL	-1.46691	7.644806	-2.66687	0.025582	-3.67709
ILMN_1664878	1510170	NLRP2	2.67701	6.805215	2.665134	0.025655	-3.67814
ILMN_2412549	1340647	GAR1	-1.05511	11.21452	-2.66472	0.025673	-3.6784
ILMN_1789505	3990646	ITPR1	2.046022	7.478286	2.664085	0.0257	-3.67878
ILMN_1668726	6100435	NFYB	-1.5972	6.686759	-2.66267	0.025759	-3.67964
ILMN_1740429	4920767	FTL	1.531468	12.42419	2.661003	0.02583	-3.68066
ILMN_1768510	4810438	MAN2B2	-1.21174	9.224062	-2.66083	0.025837	-3.68077
ILMN_1738750	780427	TFCP2	1.652824	7.418382	2.659759	0.025883	-3.68142
ILMN_1716983	3370594	LILRA2	1.098653	4.933729	2.658267	0.025947	-3.68233
ILMN_1778358	520634	ATP8A1	1.07965	5.64347	2.657893	0.025963	-3.68256
ILMN_1763347	4480523	PIK3CB	1.322757	6.16068	2.6572	0.025992	-3.68298
ILMN_1759766	360114	CTXN1	-2.14169	7.430431	-2.65712	0.025996	-3.68303
ILMN_1757072	5260156	LOC642489	1.210133	10.14095	2.656645	0.026016	-3.68332
ILMN_1808163	6110348	C11orf24	-1.246	6.97794	-2.65575	0.026054	-3.68387
ILMN_2380588	7210484	C6orf108	1.066904	9.548067	2.654492	0.026108	-3.68463
ILMN_1803464	6900521	PHTF1	1.145669	6.909047	2.65419	0.026121	-3.68482
ILMN_1735155	4560064	GLB1	1.775807	7.624613	2.652125	0.02621	-3.68608
ILMN_1909886	1710437		1.154553	6.312272	2.649778	0.026311	-3.68751
ILMN_1707339	4920053	BTG3	1.403149	9.572614	2.64954	0.026322	-3.68765
ILMN_3237385	5290592	NRBF2	1.426546	7.166291	2.64819	0.02638	-3.68848
ILMN_1682717	1190367	IER3	1.952323	6.558842	2.645216	0.02651	-3.69029
ILMN_3237256	4810750	BEND4	1.664668	7.552783	2.642174	0.026643	-3.69215
ILMN_1788118	940382	SLC23A3	-2.07417	5.566738	-2.64023	0.026728	-3.69334
ILMN_1796835	4280689	RWDD3	1.437298	6.737181	2.640014	0.026738	-3.69347
ILMN_1690761	3370048	SURF4	1.715091	8.207524	2.639965	0.02674	-3.6935
ILMN_1707137	4640161	C17orf97	-1.70352	5.651477	-2.6359	0.026919	-3.69599
ILMN_1703092	4250437	RECQL4	-1.5769	7.791633	-2.63463	0.026976	-3.69677
ILMN_1694491	3170110	CCNG1	1.076371	10.76219	2.633007	0.027048	-3.69776
ILMN_1701216	3780450	BANP	-1.14759	8.038663	-2.63285	0.027055	-3.69786
ILMN_1664863	6350131	CTRL	-1.45507	6.086571	-2.63115	0.02713	-3.6989
ILMN_1744795	2470070	TBL1X	-1.37191	9.027873	-2.63064	0.027153	-3.69921
ILMN_3250427	4210086	LOC646278	-1.16536	7.004185	-2.62954	0.027202	-3.69988
ILMN_1747251	4250136	LTB4R	-1.89792	6.903112	-2.62916	0.027219	-3.70012
ILMN_1756146	50592	WDR45	-1.3614	6.731262	-2.62879	0.027236	-3.70034
ILMN_1798705	1770397	CCNC	2.562293	8.186111	2.628181	0.027263	-3.70072
ILMN_2334242	7510470	CREB1	-1.30925	11.14078	-2.62712	0.027311	-3.70137
ILMN_1698463	1450075	ILF3	-1.09359	10.12874	-2.62608	0.027358	-3.702
ILMN_1738179	7510019	FOXP1	-1.50247	6.5768	-2.62576	0.027372	-3.7022
ILMN_1667519	6350239	RRAS2	2.426367	6.947638	2.625067	0.027403	-3.70262
ILMN_1894172	3460129		-1.96529	6.63401	-2.62453	0.027427	-3.70295
ILMN_3239343	3440441	STAG3L3	-1.66472	10.13726	-2.62271	0.02751	-3.70407
ILMN_2186806	7330053	HLA-F	2.564011	9.96821	2.62223	0.027531	-3.70436
ILMN_3176403	3850204	FTHL16	2.123761	12.12764	2.621764	0.027553	-3.70465
ILMN_2215043	2490433	RNF32	-1.15044	5.795867	-2.62066	0.027603	-3.70532
ILMN_2051684	1980369	LOC401152	-1.16356	7.90568	-2.62034	0.027617	-3.70552
ILMN_1651642	3390328	GPC2	-1.26325	8.014312	-2.61956	0.027653	-3.706
ILMN_2054607	2650598	CYP4V2	-2.00255	6.719051	-2.61862	0.027696	-3.70658
ILMN_2153916	6270274	HSPA2	1.171582	4.996196	2.616131	0.027809	-3.7081
ILMN_1769082	7330341	TXNDC5	1.245495	6.29823	2.614941	0.027864	-3.70883
ILMN_2381121	620075	UQCC	1.385895	6.674501	2.614658	0.027877	-3.70901
ILMN_2262275	1170523	TRIM13	-1.37424	9.403468	-2.61329	0.02794	-3.70985
ILMN_1753782	2810605	ZNF266	-1.01682	8.952162	-2.61322	0.027943	-3.70989

ILMN_2087692	2450465	CYBRD1	1.97283	5.667103	2.610586	0.028064	-3.71151
ILMN_2047206	1820187	TMX3	1.719766	7.037122	2.609076	0.028134	-3.71244
ILMN_2409395	2370204	CCNC	2.597814	8.360795	2.607804	0.028193	-3.71322
ILMN_1796458	3120520	GABARAPL2	1.529491	10.62134	2.60773	0.028196	-3.71326
ILMN_1809467	2630195	VAMP5	1.65393	6.657041	2.606778	0.028241	-3.71385
ILMN_1706859	990014	C22orf32	-1.20611	7.800041	-2.60591	0.028281	-3.71438
ILMN_1688381	4780192	BPTF	1.359419	6.070043	2.603232	0.028406	-3.71603
ILMN_2415979	5570593	KIAA1751	-1.52215	11.30942	-2.60199	0.028464	-3.71679
ILMN_1687896	6560121	PIK3C3	1.026007	6.770511	2.601122	0.028505	-3.71733
ILMN_1697827	1110678	ATP2A3	-1.55048	7.763469	-2.60098	0.028511	-3.71741
ILMN_3243714	3060520	LOC642073	2.309844	7.554059	2.600003	0.028557	-3.71801
ILMN_1795893	3130241	TMEM167B	1.580793	7.270157	2.599456	0.028583	-3.71835
ILMN_1695640	6560220	PTPN22	1.441606	7.715525	2.597807	0.028661	-3.71937
ILMN_1697703	6480026	HPDL	-1.27854	6.730903	-2.597	0.028699	-3.71986
ILMN_1734544	6560403	WTAP	1.613217	7.38383	2.596591	0.028718	-3.72011
ILMN_1701413	6040767	PIGQ	-1.18009	8.026801	-2.59507	0.02879	-3.72105
ILMN_1776297	2940070	GOLGA4	1.073624	5.93919	2.594104	0.028836	-3.72165
ILMN_1754579	10634	SYNE2	1.173695	6.116099	2.593823	0.028849	-3.72182
ILMN_3239135	6130576	LOC100132391	-1.22044	12.19853	-2.59364	0.028858	-3.72193
ILMN_2100693	3420358	MAP2K4	-1.22182	7.121363	-2.59333	0.028873	-3.72212
ILMN_1729546	650167	C19orf54	-1.12594	7.65722	-2.59028	0.029018	-3.724
ILMN_2077623	620546	RRAS2	2.44272	7.082103	2.58782	0.029136	-3.72552
ILMN_2148913	6280520	TMEM45A	1.052059	4.905337	2.585843	0.029231	-3.72674
ILMN_1729650	4040017	PEX7	1.290358	6.342423	2.585779	0.029234	-3.72677
ILMN_1789186	4120750	OBFC1	1.077864	8.014453	2.585543	0.029245	-3.72692
ILMN_1720513	5310079	SETBP1	-2.21496	6.938255	-2.58547	0.029249	-3.72697
ILMN_1714167	2140154	CYB5A	2.639347	9.134688	2.580649	0.029482	-3.72994
ILMN_2180239	5080328	DOPEY2	-1.70638	9.474126	-2.58034	0.029497	-3.73013
ILMN_1794333	7320687	POU2F1	-1.32916	7.920692	-2.5791	0.029557	-3.73089
ILMN_1775328	3420088	MTG1	-1.1757	6.291963	-2.57851	0.029586	-3.73126
ILMN_1699644	4670255	Mar-03	1.184476	7.172722	2.578127	0.029604	-3.7315
ILMN_1808861	7100692	LOC653803	1.115425	5.913198	2.578008	0.02961	-3.73157
ILMN_1805826	1090367	BIVM	2.077618	6.25503	2.576574	0.02968	-3.73245
ILMN_2399363	4050202	CLEC4A	1.776833	5.568801	2.576036	0.029706	-3.73279
ILMN_1770084	2940373	TACC1	1.714223	10.25272	2.575162	0.029749	-3.73333
ILMN_2380967	1510543	DNASE1L1	1.359894	6.458579	2.574578	0.029778	-3.73369
ILMN_3247482	940368	LOC100132475	-1.01875	5.597105	-2.57441	0.029786	-3.73379
ILMN_1758687	5310678	DDX39	-1.20224	6.445521	-2.5728	0.029865	-3.73478
ILMN_3251605	3610358	KLHL28	-1.35778	8.835318	-2.57211	0.029899	-3.73521
ILMN_1795822	4280300	DIS3L	-1.10431	10.09029	-2.5692	0.030043	-3.73701
ILMN_2167011	7650053	ECHDC1	1.431481	6.858365	2.569157	0.030045	-3.73704
ILMN_1698491	610528	MBD3	-1.45903	8.007129	-2.56904	0.030051	-3.73711
ILMN_1752046	6560301	SH2B3	1.05627	9.008267	2.566896	0.030157	-3.73843
ILMN_1695962	110450	SLC12A9	-1.40714	8.41041	-2.56618	0.030192	-3.73888
ILMN_1755321	60689	AAAS	-1.15403	7.350615	-2.56308	0.030347	-3.74079
ILMN_2146566	2510722	SFRS16	-1.43139	8.097196	-2.56307	0.030347	-3.7408
ILMN_1678766	6350634	DYNLT1	2.252606	8.507166	2.561797	0.030411	-3.74159
ILMN_1779648	1500192	HIST3H2A	1.087146	5.842985	2.561737	0.030414	-3.74162
ILMN_1723141	7200561	OTUD1	1.058882	6.068359	2.559495	0.030526	-3.74301
ILMN_1792931	3390273	LYSMD1	1.090409	5.91866	2.559432	0.03053	-3.74305
ILMN_1809511	6980154	GRINL1A	1.117466	5.974978	2.558708	0.030566	-3.7435
ILMN_2220403	360017	C6orf72	1.253839	7.019071	2.557256	0.030639	-3.7444
ILMN_1695853	5820403	CLK4	1.118566	6.385624	2.554303	0.030788	-3.74622

ILMN_1657451	2000468	SRPK2	1.386162	7.215603	2.552945	0.030857	-3.74707
ILMN_2285618	6520577	DIP2A	1.047415	5.879644	2.552719	0.030869	-3.74721
ILMN_1778788	6960079	AMOTL2	-1.35664	6.52995	-2.55236	0.030887	-3.74743
ILMN_1781761	3060471	ENPP4	1.703204	6.658967	2.551787	0.030916	-3.74778
ILMN_2290998	1110255	CCS	1.207524	6.340306	2.550281	0.030993	-3.74872
ILMN_1743747	670376	RUSC1	-1.06034	9.155751	-2.54897	0.03106	-3.74953
ILMN_1769245	6220746	GLIPR1	1.502324	5.989009	2.548543	0.031082	-3.74979
ILMN_1705848	6380148	FAM104B	-1.63229	6.570154	-2.54671	0.031176	-3.75093
ILMN_1658452	4060112	FAM53A	-1.05582	5.603901	-2.54566	0.03123	-3.75158
ILMN_1676058	110348	MAGOHB	-1.14618	6.597703	-2.54494	0.031266	-3.75202
ILMN_1697864	5090491	CXorf38	1.68866	8.213871	2.544706	0.031279	-3.75217
ILMN_1676528	4610674	BTN3A2	2.198456	7.892326	2.54432	0.031299	-3.75241
ILMN_1712305	2370300	CYBRD1	1.779224	5.433496	2.544214	0.031304	-3.75248
ILMN_1656285	1820008	METTL7A	-1.82954	7.409371	-2.54234	0.031401	-3.75364
ILMN_1761068	2320463	MGC52000	-1.27485	8.426957	-2.54109	0.031465	-3.75441
ILMN_1676745	150040	ZNF142	-1.09165	9.417921	-2.54072	0.031485	-3.75465
ILMN_3229859	6510669	MOBKL3	1.439479	7.269747	2.540103	0.031517	-3.75503
ILMN_2285802	4200441	SEC14L1	1.552386	7.373576	2.539482	0.031549	-3.75541
ILMN_1783676	6110095	CCDC15	-2.3073	7.606057	-2.53805	0.031624	-3.7563
ILMN_1654609	150441	TIGA1	1.156567	10.94074	2.536681	0.031695	-3.75715
ILMN_1758613	1940072	RAPGEFL1	-1.52472	5.733333	-2.5365	0.031704	-3.75726
ILMN_1655796	6760167	Mar-03	1.475816	6.996504	2.536037	0.031728	-3.75755
ILMN_1814165	6480196	SSBP3	-1.74196	6.082362	-2.53554	0.031754	-3.75786
ILMN_1696028	3890543	ETNK1	1.153654	6.614429	2.535501	0.031756	-3.75788
ILMN_1697088	2060154	ARMC5	-1.17381	6.216266	-2.53547	0.031758	-3.75791
ILMN_2400661	2100376	ZNF626	1.420402	6.133353	2.534572	0.031805	-3.75846
ILMN_1707337	290554	MSTO1	-1.31377	8.057775	-2.53426	0.031822	-3.75866
ILMN_1751636	6510445	ANKS3	-1.64259	5.96132	-2.53359	0.031857	-3.75907
ILMN_1771065	5820703	BRCA1	-1.01775	6.027295	-2.53223	0.031928	-3.75992
ILMN_1792660	6280209	CAMSAP1L1	1.097766	5.628031	2.531822	0.031949	-3.76017
ILMN_1672961	2120164	LOC652624	1.045083	12.05884	2.531219	0.031981	-3.76054
ILMN_3245380	1780189	ZNF860	-1.72883	7.478521	-2.53096	0.031995	-3.76071
ILMN_1733164	290037	FBXO11	-1.24312	8.038333	-2.52947	0.032073	-3.76163
ILMN_1800733	4230168	MANBA	1.289581	7.632222	2.529299	0.032082	-3.76174
ILMN_2229950	1580142	MPHOSPH10	-1.26607	5.956881	-2.52919	0.032088	-3.76181
ILMN_2288483	1850040	C3orf34	-1.1654	11.79633	-2.52908	0.032094	-3.76188
ILMN_1815086	7380706	NINJ1	-1.22824	8.700686	-2.52802	0.03215	-3.76253
ILMN_3235325	360292	SCARNA13	1.46579	7.650903	2.527581	0.032173	-3.76281
ILMN_2140559	1230477	IRX5	-1.22568	5.335063	-2.52708	0.0322	-3.76312
ILMN_1785528	1240441	FAM39DP	-1.54705	9.118844	-2.5251	0.032305	-3.76435
ILMN_1687484	7510088	ZFX	1.326905	6.309357	2.524697	0.032326	-3.7646
ILMN_1677951	2230273	AFF3	-1.83708	6.391484	-2.52447	0.032338	-3.76474
ILMN_1754234	1190626	ZMYND11	-2.40996	7.48643	-2.52427	0.032349	-3.76486
ILMN_1702683	4490470	SLC33A1	1.649482	6.944073	2.520065	0.032574	-3.76748
ILMN_2288070	2900129	FTO	1.130619	6.40076	2.520019	0.032576	-3.76751
ILMN_1655827	4890195	COPS2	1.519862	7.332106	2.51943	0.032608	-3.76788
ILMN_1763326	5960639	C5orf25	2.152964	6.87771	2.518427	0.032662	-3.7685
ILMN_2397721	1340634	GLB1	2.064481	8.265259	2.517448	0.032714	-3.76911
ILMN_2305721	4390687	POMT1	1.151376	6.712819	2.516792	0.03275	-3.76952
ILMN_1698231	7150224	RRM2B	1.846786	7.274537	2.516677	0.032756	-3.76959
ILMN_1707627	1940360	TPI1	2.24829	11.81981	2.515689	0.032809	-3.7702
ILMN_1655093	5490435	LOC727773	-1.10251	6.886054	-2.5141	0.032895	-3.7712
ILMN_3296923	4780692	LOC341784	-1.44051	6.532517	-2.51257	0.032978	-3.77214

ILMN_1725760	150605	YTHDC2	1.308661	6.312621	2.512306	0.032993	-3.77231
ILMN_1724158	5860053	ZNF93	1.013605	6.085137	2.512002	0.033009	-3.7725
ILMN_1780057	5810678	RENBP	1.20221	5.176394	2.511622	0.03303	-3.77274
ILMN_1705928	3170072	SNRNP200	-1.16517	7.188562	-2.50962	0.033139	-3.77398
ILMN_1753819	1430424	RFFL	1.707917	6.747849	2.506512	0.033309	-3.77592
ILMN_2282077	6580753	MIB2	-1.11188	7.441705	-2.50633	0.033319	-3.77603
ILMN_2300970	6770343	ETFB	-1.06106	9.935642	-2.50614	0.033329	-3.77616
ILMN_1763104	2650477	TRAF4	1.239732	6.482992	2.505188	0.033381	-3.77675
ILMN_2203807	7550452	MRP63	-1.50711	8.565037	-2.50456	0.033416	-3.77714
ILMN_2332795	730162	ZNF16	-1.00375	7.634544	-2.50446	0.033422	-3.7772
ILMN_3293049	5130161	LOC284167	-1.08577	8.485315	-2.50383	0.033456	-3.77759
ILMN_1654497	5260221	ATAD1	1.710572	8.069935	2.503682	0.033464	-3.77769
ILMN_1745784	7040139	ZNF324	-1.28253	7.713494	-2.50348	0.033476	-3.77781
ILMN_1784554	2900079	LOC647389	-1.66054	8.509103	-2.5026	0.033524	-3.77836
ILMN_2383150	5670709	MCHR2	-1.29684	6.14874	-2.50247	0.033531	-3.77844
ILMN_1802753	160309	TSSC4	-1.19072	8.976477	-2.50224	0.033544	-3.77858
ILMN_2344650	6550133	N4BP2L1	1.649897	6.110482	2.501395	0.033591	-3.77911
ILMN_1724493	770538	LYSMD2	2.832191	7.274296	2.501192	0.033602	-3.77924
ILMN_1759206	6840739	PLS1	1.184073	5.474338	2.500093	0.033663	-3.77992
ILMN_1807981	4220193	SIGIRR	-1.19248	7.422437	-2.49987	0.033675	-3.78006
ILMN_1778677	6480440	MRS2	1.461007	6.896361	2.499032	0.033721	-3.78059
ILMN_3235832	6560201	LOC728835	2.232852	5.994449	2.499028	0.033722	-3.78059
ILMN_2303170	2810576	MBNL3	-1.07219	6.512997	-2.49866	0.033742	-3.78082
ILMN_1672022	6650482	EPHA4	1.3538	5.448009	2.498437	0.033755	-3.78096
ILMN_1733249	1660246	GNB4	1.665989	5.673416	2.498306	0.033762	-3.78104
ILMN_1768273	3840397	POP1	1.35818	7.062836	2.498159	0.03377	-3.78113
ILMN_1690454	4200367	C3orf54	-1.17318	6.946274	-2.49789	0.033785	-3.7813
ILMN_3248324	540484	MGC16384	1.523815	7.253123	2.497326	0.033816	-3.78165
ILMN_1660462	5490068	MCOLN2	1.146965	11.2962	2.49354	0.034028	-3.78401
ILMN_1680111	4250376	AHDC1	-1.60628	6.068144	-2.49324	0.034044	-3.7842
ILMN_1778111	2850437	LOC441124	-1.03818	6.219207	-2.49268	0.034076	-3.78455
ILMN_1782241	580400	PRR12	-1.19908	5.555552	-2.49263	0.034079	-3.78458
ILMN_1760855	60324	OCRL	1.038313	6.194049	2.491664	0.034133	-3.78518
ILMN_1710954	1660220	LOC283932	-1.61595	7.121149	-2.49165	0.034134	-3.78519
ILMN_1793563	6400121	DCTN1	-1.10606	6.813807	-2.49035	0.034207	-3.786
ILMN_1784436	4850300	KIAA1688	-1.3421	5.473623	-2.4898	0.034238	-3.78635
ILMN_1801124	7200161	KIAA1826	2.00594	7.606646	2.489641	0.034247	-3.78645
ILMN_3240420	1740360	USP18	1.620874	5.386099	2.489241	0.034269	-3.7867
ILMN_1782247	4830301	KAT2A	-1.37547	9.963916	-2.48853	0.03431	-3.78714
ILMN_3238053	4210551	LOC100129211	-1.54161	10.44721	-2.48802	0.034339	-3.78746
ILMN_2049727	2810082	C20orf111	1.528806	9.214303	2.486503	0.034424	-3.78841
ILMN_1693340	5890273	RAC3	-1.35491	5.748487	-2.48622	0.03444	-3.78859
ILMN_3239766	360010	FKBP1P1	-1.63046	6.159739	-2.48603	0.034451	-3.78871
ILMN_2251766	3990040	IL1R2	1.242653	5.118552	2.485466	0.034483	-3.78906
ILMN_1679897	4150682	IGFL3	-1.04102	6.787886	-2.48344	0.034598	-3.79032
ILMN_1766798	3060747	CENTB2	1.415005	6.7394	2.483246	0.034609	-3.79045
ILMN_2384561	3120615	TJP2	1.494148	6.376104	2.483188	0.034613	-3.79048
ILMN_3249006	1660008	LOC100133888	-1.31394	8.017595	-2.4831	0.034618	-3.79054
ILMN_1801572	6330367	C19orf44	-1.21478	6.676965	-2.48279	0.034635	-3.79073
ILMN_3223500	3940521	LOC728903	-1.4403	10.95681	-2.48134	0.034718	-3.79164
ILMN_1767837	2100368	GOLT1B	1.999471	7.420693	2.481228	0.034725	-3.79171
ILMN_3225577	7320048	LOC729065	-1.14388	5.459141	-2.48116	0.034728	-3.79175
ILMN_1787657	1070133	CLDN12	1.284949	5.702509	2.48101	0.034737	-3.79184

ILMN_2198376	3420543	PSMA4	1.045226	12.06312	2.480562	0.034763	-3.79212
ILMN_1723007	940338	ZCCHC9	1.277811	9.39099	2.480515	0.034765	-3.79215
ILMN_1783852	10142	CD164	1.008797	6.710025	2.480122	0.034788	-3.7924
ILMN_1651315	5670110	HMG20B	-1.08554	9.356383	-2.47983	0.034805	-3.79258
ILMN_1793836	2140187	LOC389442	-1.76227	6.847906	-2.47946	0.034826	-3.79281
ILMN_2403852	4730703	CYTH1	2.030376	8.101296	2.479392	0.03483	-3.79286
ILMN_2383455	7400100	SUOX	1.074388	5.342143	2.479333	0.034833	-3.79289
ILMN_1778401	5310168	HLA-B	2.028829	11.1238	2.479294	0.034835	-3.79292
ILMN_2083567	6560193	PHLPP2	1.150172	6.346427	2.476418	0.035001	-3.79472
ILMN_2078724	7160747	C14orf153	-1.24248	11.37884	-2.47411	0.035134	-3.79616
ILMN_1717852	1990132	USH1G	-1.00649	6.835923	-2.47236	0.035235	-3.79725
ILMN_1658917	2260020	SLC1A1	1.173096	5.098189	2.471654	0.035276	-3.7977
ILMN_2186678	3710465	C6orf211	1.028225	6.251329	2.47082	0.035325	-3.79822
ILMN_1704972	1660010	TRIM5	1.831941	6.895502	2.469735	0.035388	-3.7989
ILMN_1808636	1510082	LARP1B	1.083335	6.110407	2.468628	0.035452	-3.79959
ILMN_1658684	2120209	ATP9B	-1.48609	6.397018	-2.46858	0.035455	-3.79962
ILMN_1730907	7000037	ZFAND1	1.118566	8.967006	2.468124	0.035482	-3.79991
ILMN_3235637	2630246	LOC100128288	-1.25449	11.34565	-2.46712	0.03554	-3.80054
ILMN_1702168	3420341	HSD17B12	1.504224	9.214526	2.466718	0.035564	-3.80079
ILMN_1709334	780296	TM9SF1	1.353558	6.626474	2.466397	0.035583	-3.80099
ILMN_1801119	3180494	BCL2	1.868231	7.290171	2.464856	0.035673	-3.80196
ILMN_1794070	5910273	ANKRD13B	-1.24015	5.484965	-2.46447	0.035696	-3.8022
ILMN_2078547	1240576	HSPC268	-1.42386	10.67663	-2.46429	0.035706	-3.80231
ILMN_2083559	1570685	KIAA0415	-1.31173	5.838757	-2.46255	0.035809	-3.8034
ILMN_3236061	630017	ZNF783	-1.03664	7.026121	-2.46157	0.035867	-3.80402
ILMN_2211922	5050750	PPP4R1L	-1.01027	5.806502	-2.46061	0.035923	-3.80461
ILMN_1764415	1230497	ZNF585A	1.651711	7.359685	2.460394	0.035936	-3.80475
ILMN_1806032	4260689	SLC25A16	-1.20711	6.795865	-2.45971	0.035977	-3.80518
ILMN_1813641	1110139	C9orf167	1.009389	5.818804	2.458992	0.036019	-3.80563
ILMN_3235584	3840110	TRIM66	-1.47128	7.820762	-2.45721	0.036125	-3.80675
ILMN_1806862	7570056	FLJ35282	-1.35039	5.062065	-2.45686	0.036146	-3.80697
ILMN_1733155	2640601	GIT1	-1.52672	7.279793	-2.45678	0.03615	-3.80702
ILMN_1745533	1400259	FAM117A	1.102367	6.112267	2.456637	0.036159	-3.80711
ILMN_1789106	2100519	IPP	-1.62888	7.194306	-2.45531	0.036238	-3.80794
ILMN_2246956	4150201	BCL2	2.11007	7.468717	2.455277	0.03624	-3.80796
ILMN_2396996	2570725	PPCS	1.325152	8.438429	2.455036	0.036254	-3.80811
ILMN_3251728	3120452	MTMR10	1.485952	7.678631	2.454592	0.036281	-3.80839
ILMN_1709800	6290678	POMZP3	1.003908	5.340654	2.453963	0.036318	-3.80879
ILMN_2358980	5720487	ILK	1.202575	6.709902	2.453718	0.036333	-3.80894
ILMN_2074044	6330187	PLS1	1.561091	6.366337	2.453337	0.036356	-3.80918
ILMN_1795846	6270647	PPP2R5C	-1.27143	5.717861	-2.45145	0.036469	-3.81036
ILMN_1797031	6420142	HSPBAP1	-1.21412	7.560229	-2.44948	0.036587	-3.8116
ILMN_1658821	1440438	SAMD1	-1.27537	7.524006	-2.44854	0.036644	-3.81219
ILMN_2312275	6860553	SRP54	1.096734	10.31019	2.448089	0.036671	-3.81247
ILMN_1664978	2470093	TJP2	1.506602	8.058931	2.446857	0.036746	-3.81325
ILMN_1740180	6100523	SNX3	1.310354	11.18123	2.445957	0.0368	-3.81381
ILMN_1652846	4070379	PCYT2	-1.6223	7.640863	-2.44449	0.036889	-3.81474
ILMN_2099249	2260070	MTERF	-1.20293	8.146428	-2.44315	0.036971	-3.81558
ILMN_2413808	460102	CD53	1.592008	9.181733	2.442919	0.036985	-3.81572
ILMN_2398432	4570102	BRMS1	-1.07497	10.75073	-2.44187	0.037049	-3.81638
ILMN_2175075	7200037	SFRS4	1.164872	10.85297	2.441367	0.037079	-3.81669
ILMN_1827211	3890414		-1.30949	6.714401	-2.44061	0.037125	-3.81717
ILMN_1872419	2030020		-1.3311	10.74719	-2.44052	0.037131	-3.81723

ILMN_2370882	620577	ACSL5	1.152696	6.520354	2.440372	0.03714	-3.81732
ILMN_3240177	3190274	LLPH	-1.17561	10.21008	-2.44013	0.037155	-3.81747
ILMN_1752451	4670162	CTSH	1.712848	6.449687	2.439543	0.037191	-3.81784
ILMN_1811648	3890670	DCAKD	-1.00384	10.24389	-2.43827	0.037268	-3.81864
ILMN_1799725	10747	DOCK2	1.447633	10.77525	2.438072	0.037281	-3.81877
ILMN_1708632	3840278	ZNF771	-1.5327	6.086212	-2.43806	0.037282	-3.81878
ILMN_3237404	1240669	LOC100132585	-1.48651	11.20841	-2.43725	0.037332	-3.81928
ILMN_1673980	6130195	LOC643669	-1.47324	5.992774	-2.43723	0.037333	-3.8193
ILMN_1660793	3460132	PAQR4	-1.16691	9.792315	-2.4371	0.03734	-3.81937
ILMN_1802106	1190026	APOBEC3G	2.018873	7.780311	2.436764	0.037361	-3.81959
ILMN_1811050	4490537	CCDC88A	-1.44398	7.839155	-2.43641	0.037383	-3.81981
ILMN_2407314	7550386	CYP26A1	-1.7393	5.255715	-2.43466	0.037491	-3.82091
ILMN_2217329	1300743	IAH1	1.452007	8.891459	2.433993	0.037532	-3.82133
ILMN_2298511	1740717	BOLA2	-1.25062	9.128711	-2.43383	0.037542	-3.82143
ILMN_3237177	990332	FAM175A	-1.47453	11.31512	-2.43378	0.037545	-3.82147
ILMN_1710906	2260603	RNF145	1.866426	7.252974	2.43149	0.037687	-3.8229
ILMN_1797342	5270435	FNBP1	1.770177	10.62611	2.430823	0.037728	-3.82332
ILMN_1693669	2260630	WDR79	-1.70072	7.471128	-2.42878	0.037855	-3.82461
ILMN_2117171	7210450	LMO4	-1.169	11.4153	-2.4276	0.037929	-3.82535
ILMN_1723689	5570091	RANBP3	-1.04246	7.438647	-2.42719	0.037955	-3.82561
ILMN_1680805	5810019	IL28RA	-1.48192	7.53319	-2.42717	0.037956	-3.82563
ILMN_1679912	6250397	DPH3	1.021891	7.667441	2.426607	0.037991	-3.82598
ILMN_1676423	4230653	CCNC	1.873774	8.290973	2.42412	0.038147	-3.82754
ILMN_1806908	5090563	PRKCB1	1.410833	10.34731	2.423085	0.038212	-3.82819
ILMN_3251587	6290142	LOC100008589	1.255677	12.3411	2.422829	0.038228	-3.82836
ILMN_3243069	4670561	LOC100133692	-1.53127	7.818143	-2.4221	0.038273	-3.82881
ILMN_1745043	6060470	LDHC	1.250281	5.220168	2.41973	0.038423	-3.83031
ILMN_1714327	2510674	SETD1A	-1.27748	8.85905	-2.41848	0.038502	-3.83109
ILMN_1777998	3850148	ARHGAP25	-1.67312	7.845315	-2.41814	0.038524	-3.83131
ILMN_1853631	4920451		-1.48117	6.525664	-2.41688	0.038604	-3.8321
ILMN_2215824	6450661	ANKRD20A1	-2.32792	7.375566	-2.4156	0.038685	-3.83291
ILMN_1669113	1570484	ATF5	-2.14534	10.54601	-2.41292	0.038856	-3.8346
ILMN_2390853	1980288	CTSH	1.848809	9.427482	2.412372	0.038891	-3.83494
ILMN_1754211	430044	DCLRE1C	-1.09752	7.693169	-2.41178	0.038929	-3.83532
ILMN_1714349	2350524	GLCE	1.230756	6.033627	2.411119	0.038971	-3.83573
ILMN_1674941	7040674	ANO6	1.804275	7.363664	2.41092	0.038984	-3.83586
ILMN_3235185	3830537	SNRNP200	-1.33812	7.66425	-2.41037	0.039019	-3.8362
ILMN_3244583	4070376	NCRNA00219	1.096192	10.81821	2.410223	0.039029	-3.8363
ILMN_1782331	70162	TDG	-1.3776	10.07114	-2.40977	0.039058	-3.83659
ILMN_1763452	7050152	EVI2B	2.046315	8.276653	2.408874	0.039116	-3.83715
ILMN_1729318	20403	TOR1AIP1	1.578688	8.13271	2.408846	0.039117	-3.83717
ILMN_1777261	780332	FAM3C	1.82964	7.166473	2.408582	0.039134	-3.83733
ILMN_1812191	3130291	C12orf57	2.300657	10.02401	2.408009	0.039171	-3.83769
ILMN_1722538	5670162	DEFB123	-1.12226	5.691338	-2.40739	0.039211	-3.83808
ILMN_3296519	1010064	LOC728002	-1.25284	7.251701	-2.40712	0.039229	-3.83826
ILMN_1657011	3180468	LOC286208	-1.78271	8.72988	-2.40557	0.039329	-3.83923
ILMN_1731745	6860278	NINJ2	1.626023	5.982064	2.404178	0.039419	-3.84011
ILMN_1661490	780475	PFDN6	-1.08095	8.754041	-2.404	0.03943	-3.84022
ILMN_1703593	5700343	BAIAP2L1	1.732175	5.189915	2.402753	0.039511	-3.84101
ILMN_1728073	1240674	DENND1A	1.14405	5.928613	2.402406	0.039534	-3.84123
ILMN_3275106	4890358	LOC100131866	1.541206	9.536724	2.401713	0.039579	-3.84167
ILMN_1707481	5700020	BTBD15	-1.04286	7.779013	-2.40169	0.039581	-3.84168
ILMN_1776314	1030468	CHRNA10	-1.35683	5.76545	-2.40129	0.039606	-3.84193

ILMN_1799389	1470092	FBXL6	-1.18639	7.192949	-2.40082	0.039637	-3.84223
ILMN_2347424	4560019	MBOAT2	-1.62686	6.79004	-2.40076	0.039641	-3.84227
ILMN_2134538	460164	FTHL11	1.226216	7.826451	2.399651	0.039714	-3.84297
ILMN_1796949	6290358	TPX2	1.316183	8.887473	2.399288	0.039737	-3.8432
ILMN_1700834	4060021	SLK	1.336487	6.931703	2.396959	0.03989	-3.84467
ILMN_1732534	7650347	CHMP5	1.605407	8.889989	2.396468	0.039922	-3.84498
ILMN_1760575	6180414	PTP4A1	1.390966	6.638737	2.396078	0.039948	-3.84522
ILMN_2382290	3940195	KREMEN2	-1.64834	7.38627	-2.39604	0.03995	-3.84525
ILMN_1773395	110326	RDH5	-1.35322	6.512702	-2.396	0.039953	-3.84527
ILMN_3275275	4150047	LOC727962	-1.41712	7.502383	-2.3959	0.039959	-3.84534
ILMN_1770338	2680110	TM4SF1	1.34233	5.388691	2.39452	0.04005	-3.84621
ILMN_1789492	4590154	ZDHHC8	-1.09341	10.87534	-2.39448	0.040053	-3.84624
ILMN_2261076	6180048	NEDD9	1.785392	5.937889	2.394419	0.040057	-3.84627
ILMN_1740385	1070376	CEP164	-1.53864	7.073395	-2.39408	0.040079	-3.84648
ILMN_1675640	7040035	OAS1	1.627002	7.172236	2.39379	0.040098	-3.84667
ILMN_1759792	3310672	CLIP4	1.164749	5.061494	2.392815	0.040163	-3.84729
ILMN_2370464	4860367	ATRIP	-1.4142	8.166661	-2.38918	0.040403	-3.84958
ILMN_2096970	2940450	MYO3B	-1.55736	7.966837	-2.38878	0.04043	-3.84983
ILMN_2396272	2570433	PDCD4	-1.01153	11.68497	-2.38798	0.040483	-3.85034
ILMN_1723969	1570079	PLCB1	-2.03761	5.781186	-2.38679	0.040563	-3.85109
ILMN_1664168	2480475	SLC25A11	-1.63627	7.933065	-2.38549	0.040649	-3.85192
ILMN_2154322	1580608	SEMA3E	-1.34833	10.43502	-2.38394	0.040753	-3.85289
ILMN_2381197	7160010	RNF19A	1.505155	7.291352	2.383272	0.040798	-3.85332
ILMN_1706610	3930215	PLEKHA9	-1.07628	8.087894	-2.38208	0.040878	-3.85407
ILMN_1758633	3830538	CCDC130	-1.09035	10.28767	-2.38205	0.04088	-3.85409
ILMN_1810267	3290392	DNHD1	-1.17018	5.818188	-2.38195	0.040887	-3.85415
ILMN_1743476	840195	LOC653829	-1.98609	8.489767	-2.38158	0.040912	-3.85439
ILMN_2285713	2060110	TDP1	-1.4225	10.6216	-2.38071	0.04097	-3.85494
ILMN_1675435	50070	ANKRD16	-1.13804	6.345769	-2.38005	0.041015	-3.85536
ILMN_1763852	2230678	ACACB	-2.1718	7.143367	-2.37981	0.041031	-3.85551
ILMN_2081398	2450037	KIF3B	1.212872	6.269425	2.378463	0.041122	-3.85636
ILMN_1742853	1400300	LOC652184	1.523915	5.674365	2.378348	0.04113	-3.85643
ILMN_2219437	2230114	PRRG4	-1.41899	7.723227	-2.37801	0.041152	-3.85664
ILMN_1742541	4220161	ZNF518A	1.646378	7.522138	2.377961	0.041156	-3.85668
ILMN_1662843	520678	CD53	1.615562	8.582289	2.377317	0.041199	-3.85708
ILMN_2376458	2900053	CSF2RA	-1.17809	11.93172	-2.37677	0.041237	-3.85743
ILMN_3187680	3390379	ACCS	-2.7355	6.499257	-2.3755	0.041323	-3.85823
ILMN_1763972	5260309	TJP2	1.194383	6.025889	2.374647	0.041381	-3.85877
ILMN_2215382	4390661	DDX51	-1.14913	12.11123	-2.37354	0.041456	-3.85948
ILMN_2371379	4200259	ACLY	-1.09449	10.69008	-2.37273	0.041512	-3.85999
ILMN_1724822	5670091	LOC399744	-1.1493	6.037205	-2.37161	0.041588	-3.86069
ILMN_1682197	1230731	NFXL1	1.348355	7.590281	2.370631	0.041655	-3.86132
ILMN_3243302	6420333	C8orf30B	-1.27379	6.417298	-2.3695	0.041732	-3.86203
ILMN_1781468	3930390	SMAP2	1.295906	7.672375	2.369326	0.041744	-3.86214
ILMN_1786469	4880685	FBXO22	1.372835	7.501035	2.369137	0.041757	-3.86226
ILMN_1690806	4560270	PTPLB	1.369583	8.246717	2.369033	0.041764	-3.86233
ILMN_3251415	7560296	RBM43	-1.11003	5.736251	-2.36891	0.041773	-3.86241
ILMN_1859657	5290653		-1.14039	6.347109	-2.36833	0.041813	-3.86277
ILMN_1653134	5340148	TMEM188	1.448707	7.757236	2.367605	0.041863	-3.86323
ILMN_1757827	870692	ECOP	1.416112	7.488323	2.365752	0.04199	-3.86441
ILMN_1708382	4210102	C3orf75	-1.12247	9.664196	-2.36533	0.04202	-3.86467
ILMN_1662839	6650324	PLEKHA1	1.811547	6.495165	2.365219	0.042027	-3.86474
ILMN_1813975	6620278	ADI1	-1.22664	6.709573	-2.36504	0.04204	-3.86486

ILMN_2388800	7050575	PPAP2B	1.151434	5.306729	2.364787	0.042057	-3.86502
ILMN_1762312	1240309	FOXRED1	-1.00658	9.59191	-2.36449	0.042077	-3.8652
ILMN_1731783	3370164	ATP1A1	1.041673	8.952455	2.363511	0.042145	-3.86582
ILMN_1723912	3870338	IFI44L	3.990787	7.507588	2.362689	0.042202	-3.86635
ILMN_2092232	7400544	TSR1	-1.60737	5.369778	-2.36251	0.042215	-3.86646
ILMN_1726391	6940025	MRPL39	1.354943	10.00066	2.362032	0.042248	-3.86676
ILMN_1709124	2900438	ANO8	-1.22117	5.744882	-2.36099	0.04232	-3.86742
ILMN_1678612	2490598	ANXA6	1.461877	5.991277	2.360954	0.042323	-3.86744
ILMN_1756590	5090097	SYS1	1.035638	6.319954	2.359583	0.042418	-3.86831
ILMN_2378952	6520128	GPX4	1.367448	11.88643	2.359512	0.042423	-3.86836
ILMN_1801121	2940349	SENP2	1.61038	7.162551	2.356736	0.042617	-3.87012
ILMN_2415776	6450189	WWOX	-1.70803	7.411659	-2.3567	0.04262	-3.87014
ILMN_2364272	990128	MBNL2	1.423228	7.247894	2.35662	0.042625	-3.87019
ILMN_1713603	2190411	PRKCB1	1.890316	7.177925	2.356229	0.042653	-3.87044
ILMN_1709439	5900332	CHMP1A	-1.13908	8.786316	-2.35589	0.042676	-3.87065
ILMN_2243516	1740767	C11orf63	-1.48449	7.854861	-2.35553	0.042702	-3.87088
ILMN_3257475	1230064	LOC100129866	1.088332	6.40484	2.354407	0.042781	-3.8716
ILMN_1880280	1500309		-2.06601	8.549902	-2.35417	0.042798	-3.87175
ILMN_2363439	770372	RYK	1.145939	6.200651	2.35305	0.042876	-3.87246
ILMN_3278506	2480041	LOC148430	1.565534	11.40453	2.351691	0.042972	-3.87332
ILMN_1750521	2570504	UCP3	-1.13497	5.364246	-2.35016	0.04308	-3.87429
ILMN_2367428	6760202	FAM96A	1.487929	9.039686	2.348947	0.043166	-3.87506
ILMN_3262849	460452	LOC100128510	-1.55321	10.01756	-2.34873	0.043181	-3.87519
ILMN_2286014	2600273	CATSPER2	-1.11606	11.85802	-2.34819	0.04322	-3.87554
ILMN_3182120	6620575	LOC100129522	-1.07489	5.845331	-2.34794	0.043238	-3.8757
ILMN_3247139	6250427	C17orf96	-1.00928	7.575388	-2.34769	0.043255	-3.87586
ILMN_1673804	5820719	ZNF426	1.126771	5.903051	2.347572	0.043264	-3.87593
ILMN_1884723	780682		-1.1887	5.74889	-2.34645	0.043344	-3.87664
ILMN_1653039	7040482	LOC642934	-1.08344	10.16096	-2.34609	0.043369	-3.87687
ILMN_1720482	5560162	CEND1	-1.30105	6.117152	-2.34603	0.043373	-3.87691
ILMN_3239603	3930068	LOC727967	-1.42519	6.425175	-2.34568	0.043399	-3.87713
ILMN_1710284	1010528	HES1	1.157563	5.269067	2.344779	0.043463	-3.8777
ILMN_2086077	7550500	JUNB	1.455408	5.925284	2.344234	0.043502	-3.87805
ILMN_1662587	5050577	PNPLA7	-1.1667	6.746914	-2.34418	0.043506	-3.87809
ILMN_2168217	4040037	EBI2	2.203455	6.371317	2.343144	0.04358	-3.87874
ILMN_1782954	1300482	HIP2	1.208951	8.418877	2.342347	0.043637	-3.87925
ILMN_1757074	450348	GNG10	2.122028	7.736126	2.341893	0.04367	-3.87953
ILMN_1758543	4070088	CNIH	1.546973	10.06788	2.33989	0.043813	-3.88081
ILMN_1750160	3990465	FASTKD3	1.289082	8.35176	2.339523	0.04384	-3.88104
ILMN_1720285	2680471	ESD	1.305487	11.24513	2.338668	0.043901	-3.88158
ILMN_1744963	4780671	ERO1L	1.298289	7.171867	2.338528	0.043912	-3.88167
ILMN_1729180	3940292	GATM	1.902983	5.517555	2.337401	0.043993	-3.88239
ILMN_1683811	3360241	TNPO3	-1.07357	8.854918	-2.33652	0.044057	-3.88295
ILMN_2120982	5550647	ALG11	-1.71691	5.746888	-2.33649	0.044059	-3.88296
ILMN_2098616	7330523	C5orf39	-1.28183	7.329887	-2.33596	0.044097	-3.8833
ILMN_2346137	5360707	ZNF557	-1.57999	8.774595	-2.3349	0.044174	-3.88397
ILMN_1716086	150070	WDR33	1.690634	7.457115	2.333962	0.044242	-3.88457
ILMN_1725518	5050707	ANGPTL6	1.399463	6.964479	2.333838	0.044251	-3.88465
ILMN_2373763	5290465	CASP7	1.190665	6.324334	2.333776	0.044256	-3.88469
ILMN_1652147	6620181	MRPL43	-1.1867	6.126969	-2.33377	0.044256	-3.8847
ILMN_1673548	1740435	HSPC159	1.00399	5.345691	2.333401	0.044283	-3.88493
ILMN_1784780	5550600	CIC	-1.13448	7.295036	-2.33283	0.044325	-3.88529
ILMN_3238576	5900270	FOXD4L4	-1.29575	6.028166	-2.33275	0.04433	-3.88534

ILMN_2055310	240364	MBD4	-1.39771	10.92794	-2.33132	0.044435	-3.88625
ILMN_1732127	4200209	RBKS	-1.01275	6.046617	-2.33089	0.044466	-3.88652
ILMN_2091412	830324	FLT3LG	-1.12568	5.96362	-2.33077	0.044475	-3.8866
ILMN_1669210	1430427	SNORD16	1.418288	7.143921	2.330688	0.044481	-3.88665
ILMN_2059173	2900307	SLC35E1	-1.32819	10.54694	-2.33033	0.044507	-3.88688
ILMN_1676241	6760273	BCOR	-1.25835	7.344352	-2.32902	0.044603	-3.88771
ILMN_1685574	7100215	TSC22D2	1.163523	6.982339	2.328941	0.044609	-3.88776
ILMN_1660864	510092	RHBDL1	-1.61414	5.854703	-2.3287	0.044626	-3.88791
ILMN_1715188	5360528	USP8	1.44109	6.923692	2.328649	0.04463	-3.88795
ILMN_1780397	3450154	TRAF3IP3	1.461046	6.125115	2.327453	0.044718	-3.88871
ILMN_2299612	4780647	TMEM150A	-1.04134	6.133332	-2.3269	0.044759	-3.88906
ILMN_1788547	1990577	GCLM	1.56521	7.334213	2.326875	0.04476	-3.88908
ILMN_3290035	6450524	LOC653737	1.367205	8.961251	2.326717	0.044772	-3.88918
ILMN_1727348	5360204	NMD3	1.796722	7.971001	2.326547	0.044784	-3.88928
ILMN_1696601	6980100	VARS	-1.10444	10.21308	-2.32631	0.044802	-3.88943
ILMN_2139351	6980435	ZNF232	-1.33504	8.327695	-2.3263	0.044803	-3.88944
ILMN_1692486	4060255	ZNRD1	1.299569	9.188386	2.326125	0.044815	-3.88955
ILMN_1758864	7100161	VNN2	1.382313	5.981355	2.325377	0.044871	-3.89003
ILMN_1685079	240750	TELO2	-1.26923	6.640708	-2.32423	0.044955	-3.89075
ILMN_1912662	5340719		-1.34632	9.383761	-2.32385	0.044983	-3.891
ILMN_1721116	1740139	USP10	1.934726	8.336754	2.322143	0.04511	-3.89208
ILMN_2107004	3870386	GPR1	-1.59455	9.859468	-2.31995	0.045273	-3.89348
ILMN_1717973	5910082	TMEM1	-1.61489	6.378659	-2.31973	0.045289	-3.89362
ILMN_1737343	2450563	FNIP1	1.587052	7.630758	2.319619	0.045297	-3.89369
ILMN_1683096	1820347	ASB1	-1.33054	8.119332	-2.31924	0.045325	-3.89393
ILMN_1820295	1510280		-1.94801	7.03474	-2.31834	0.045392	-3.8945
ILMN_1714352	3850731	DMWD	-1.02161	7.580246	-2.31752	0.045453	-3.89503
ILMN_1689156	2140291	MMAB	-1.14065	6.697751	-2.31738	0.045464	-3.89511
ILMN_1730678	1770273	ANKRD30B	-1.01289	12.1152	-2.31723	0.045475	-3.89521
ILMN_1714417	4040605	PSEN2	1.091521	6.371937	2.315531	0.045602	-3.89629
ILMN_1735014	4040097	KLF6	1.234849	9.329122	2.315161	0.04563	-3.89652
ILMN_1741356	1770224	PRICKLE1	-1.5999	8.216658	-2.31487	0.045652	-3.89671
ILMN_1779252	5960747	TRIM22	2.181337	7.558491	2.314633	0.045669	-3.89686
ILMN_1651415	6270408	DERL3	-1.1653	6.627897	-2.31284	0.045804	-3.898
ILMN_3283592	4050414	LOC442609	-1.40136	6.635329	-2.31191	0.045874	-3.89859
ILMN_2249473	430669	SPTLC1	-1.00648	12.396	-2.3107	0.045965	-3.89936
ILMN_1722025	1500131	CPEB4	1.094012	5.546331	2.310634	0.04597	-3.89941
ILMN_1706498	7210170	DSE	1.867453	5.540231	2.310449	0.045984	-3.89952
ILMN_1789074	6380717	HSPA1A	1.172436	4.998584	2.31039	0.045989	-3.89956
ILMN_1799487	620112	N4BP2L1	1.540835	5.915151	2.310264	0.045998	-3.89964
ILMN_1664153	4810451	SLC30A5	1.208711	7.667848	2.31005	0.046014	-3.89978
ILMN_2076463	160279	SLC15A4	2.138525	9.277019	2.309613	0.046047	-3.90006
ILMN_1662049	830719	AGPAT5	2.284683	7.99369	2.309606	0.046048	-3.90006
ILMN_1731619	4220181	DAD1	2.061302	11.01056	2.309339	0.046068	-3.90023
ILMN_1781803	3120554	FIZ1	-1.34326	7.031755	-2.30883	0.046107	-3.90056
ILMN_2154052	1740242	FVT1	1.464807	7.612863	2.308216	0.046153	-3.90095
ILMN_1800311	130121	HSF2	1.478561	7.59374	2.308063	0.046165	-3.90104
ILMN_1808566	3870193	TMEM180	-1.44415	6.39079	-2.30771	0.046192	-3.90127
ILMN_1800420	6940600	RNF214	1.03167	6.028665	2.306765	0.046263	-3.90187
ILMN_2346831	2060079	MGAT2	1.647893	7.309739	2.305869	0.046331	-3.90244
ILMN_2105033	3180746	PLDN	-1.28591	11.01474	-2.30574	0.046341	-3.90252
ILMN_1915553	460689		-1.6028	6.492795	-2.30559	0.046352	-3.90262
ILMN_1722292	5700112	AVL9	1.125014	6.077838	2.305567	0.046354	-3.90263

ILMN_2101651	4890703	MBTD1	-1.67798	8.358303	-2.30548	0.046361	-3.90269
ILMN_1736729	240722	OAS2	1.46738	6.375393	2.304436	0.04644	-3.90335
ILMN_1765532	1820538	RDBP	-1.2889	8.114643	-2.30341	0.046519	-3.90401
ILMN_1695092	3390370	WRB	1.923944	7.462226	2.302393	0.046596	-3.90465
ILMN_2319910	3170020	DGKA	-1.03418	7.459787	-2.30102	0.046702	-3.90553
ILMN_1763640	1990762	KIAA1602	-1.12619	6.393926	-2.30094	0.046707	-3.90558
ILMN_2121068	5260546	ADAM17	-1.55316	8.647799	-2.30068	0.046727	-3.90574
ILMN_2388547	5700725	EPSTI1	2.883293	7.843229	2.300376	0.046751	-3.90594
ILMN_2287147	5490187	UBTF	-1.07832	5.617616	-2.29905	0.046852	-3.90678
ILMN_1681984	3610202	GALNT10	1.399143	6.295833	2.299028	0.046854	-3.9068
ILMN_2192683	4260142	DHX37	-1.08629	9.501129	-2.29892	0.046863	-3.90687
ILMN_1722492	5260195	DUSP19	-1.11786	11.75797	-2.29864	0.046884	-3.90704
ILMN_2117569	3870450	DEM1	-1.24287	10.59735	-2.29837	0.046905	-3.90722
ILMN_3304396	3520259	LOC730202	-1.87903	7.010698	-2.29793	0.046939	-3.9075
ILMN_2366334	7050736	FERMT3	1.37711	7.121328	2.29777	0.046951	-3.9076
ILMN_1810941	520446	COMT	1.030047	5.773695	2.29656	0.047044	-3.90837
ILMN_1700466	6480626	LOC389517	-1.36143	5.704644	-2.29628	0.047066	-3.90855
ILMN_1683792	3290292	LAP3	2.1975	9.186658	2.296145	0.047077	-3.90863
ILMN_1782110	6060326	ZNF295	1.260349	6.928035	2.295517	0.047125	-3.90903
ILMN_1680145	5130095	SART1	-1.03489	5.317782	-2.29525	0.047146	-3.90921
ILMN_1666078	1980592	HLA-H	1.804916	8.130883	2.294882	0.047174	-3.90944
ILMN_2263466	610093	ACADVL	1.105641	5.701404	2.29484	0.047178	-3.90947
ILMN_1652128	2510592	LMBRD1	1.569701	9.217609	2.294668	0.047191	-3.90958
ILMN_2110751	6280731	CHRNA5	-1.27538	10.7674	-2.29321	0.047304	-3.91051
ILMN_2105253	6220202	PTGR2	-1.62827	8.746042	-2.29235	0.047371	-3.91105
ILMN_2190779	6900014	PHAX	-1.57131	7.735354	-2.29213	0.047387	-3.91119
ILMN_2364852	2570477	BTN2A1	1.643228	7.810743	2.291964	0.047401	-3.9113
ILMN_1874016	1030608		-1.14366	5.833765	-2.29157	0.047432	-3.91155
ILMN_1730523	5690132	FAM195A	2.018966	8.065354	2.290805	0.047491	-3.91204
ILMN_1677113	6840523	RNF8	1.65124	7.07019	2.290711	0.047498	-3.9121
ILMN_2205470	4780121	FAM153B	-1.90891	8.238298	-2.29046	0.047518	-3.91226
ILMN_1766115	3890152	PLEKHF2	-1.0631	7.029346	-2.29035	0.047526	-3.91233
ILMN_3261439	3610427	LOC100128098	-1.53152	10.78522	-2.28995	0.047557	-3.91258
ILMN_1829989	990600		1.052522	6.557621	2.289639	0.047582	-3.91278
ILMN_3234384	6940259	LOC728953	-1.77823	7.785559	-2.28886	0.047643	-3.91328
ILMN_1762426	2480768	DHFRL1	-1.46078	7.732712	-2.28819	0.047695	-3.9137
ILMN_2072140	1170717	BTF3L4	1.921738	7.884468	2.286476	0.047829	-3.9148
ILMN_1742379	4210463	IFT122	-1.45612	7.629756	-2.28603	0.047864	-3.91508
ILMN_1702125	2470328	HOXB7	1.332348	6.007618	2.284557	0.04798	-3.91602
ILMN_1712755	6590088	LRRC41	1.025571	8.553228	2.284226	0.048006	-3.91623
ILMN_1682428	1710242	C1orf59	2.280104	8.256987	2.284132	0.048014	-3.91629
ILMN_1704550	3990523	AZIN1	1.401462	6.772826	2.284025	0.048022	-3.91636
ILMN_1809750	2470722	TDRD1	-1.46247	8.270218	-2.28399	0.048025	-3.91639
ILMN_1711078	2640246	CDC2L2	-1.80883	7.705597	-2.2839	0.048032	-3.91644
ILMN_1664012	5860358	CANT1	1.537334	7.279501	2.283443	0.048068	-3.91673
ILMN_1781943	150543	FAM83D	2.038637	7.695168	2.282865	0.048114	-3.9171
ILMN_2311166	2490411	ITGB5	1.160605	4.940905	2.282589	0.048135	-3.91728
ILMN_1738759	3840689	PIGT	1.362652	7.595403	2.282513	0.048141	-3.91733
ILMN_2181432	5910349	SPC24	-1.16495	10.59308	-2.28246	0.048146	-3.91736
ILMN_2175094	7150184	TDRD1	-1.41331	8.614856	-2.28232	0.048157	-3.91745
ILMN_1716276	2970575	CCL4L2	1.954399	6.020604	2.282245	0.048163	-3.9175
ILMN_3229467	6380259	LOC729217	2.159228	8.785019	2.281694	0.048206	-3.91785
ILMN_1727315	60524	DENND1A	1.783644	7.162744	2.281397	0.04823	-3.91804

ILMN_1666827	1470519	C4orf41	1.186558	6.272702	2.281017	0.04826	-3.91828
ILMN_1681845	10050	PAPD4	2.091558	8.812523	2.278827	0.048433	-3.91968
ILMN_2189222	7100349	KLHL8	-1.19412	9.376838	-2.27872	0.048442	-3.91974
ILMN_1685676	3830682	TTC9C	1.17385	6.204226	2.278587	0.048452	-3.91983
ILMN_1673936	7000470	KHSRP	-1.00416	10.49487	-2.27777	0.048517	-3.92035
ILMN_1656111	4830424	MYLIP	2.44185	8.491021	2.277115	0.04857	-3.92077
ILMN_1889527	1190484		-1.0486	5.366089	-2.27639	0.048628	-3.92124
ILMN_2054145	1990575	PAK1IP1	-1.53505	8.691786	-2.27578	0.048676	-3.92162
ILMN_1761086	6060379	VPS54	1.234435	6.316897	2.275627	0.048688	-3.92172
ILMN_1657898	6450184	MTP18	1.33768	6.4799	2.275265	0.048717	-3.92195
ILMN_1671742	70041	UPF3A	-1.43877	9.709402	-2.27522	0.04872	-3.92198
ILMN_1721339	3190138	ZNF276	-1.12359	5.789569	-2.27366	0.048846	-3.92298
ILMN_1860168	6560743		-1.12957	5.788454	-2.27312	0.048889	-3.92332
ILMN_2361807	1030593	OS9	1.076517	6.008105	2.271282	0.049036	-3.92449
ILMN_1809141	1430767	ING4	-1.06461	7.064757	-2.27122	0.049041	-3.92453
ILMN_1767360	5050368	IL10RB	1.353139	7.042749	2.270968	0.049062	-3.92469
ILMN_1803348	7650309	EHBP1	1.673523	8.046323	2.27067	0.049086	-3.92489
ILMN_3236599	7610553	FLJ44054	-1.13754	6.838142	-2.27023	0.049121	-3.92517
ILMN_1806692	3800139	HEXB	1.313149	8.935555	2.270091	0.049132	-3.92526
ILMN_2053490	1690600	FAM53B	-1.05877	6.211942	-2.27009	0.049132	-3.92526
ILMN_1652790	1690121	CLK1	1.237348	6.456121	2.268716	0.049243	-3.92613
ILMN_1660787	5220220	SUCLA2	1.645806	7.737657	2.268536	0.049258	-3.92625
ILMN_3236036	7570184	LOC283663	1.21296	5.471041	2.26802	0.049299	-3.92658
ILMN_1756696	7210382	USF2	-1.02782	8.591107	-2.26772	0.049323	-3.92677
ILMN_1652309	840484	TTC8	-1.51657	5.664338	-2.26731	0.049357	-3.92703
ILMN_1697268	1190142	EMILIN2	1.19252	5.193321	2.267089	0.049375	-3.92717
ILMN_2349138	4480192	CDC42SE1	1.596004	7.413876	2.266972	0.049384	-3.92725
ILMN_1802905	1470315	PIAS4	-1.06792	9.461761	-2.26686	0.049394	-3.92732
ILMN_1673676	630100	SNX5	2.297844	8.952859	2.266533	0.04942	-3.92753
ILMN_1703697	2450612	LANCL1	1.463951	8.926946	2.264841	0.049557	-3.92861
ILMN_1696432	3180048	IDH1	1.377301	6.864004	2.264646	0.049573	-3.92873
ILMN_1768357	5670195	RTN1	-1.22676	5.658892	-2.26442	0.049591	-3.92888
ILMN_2307740	2120451	CD46	2.182516	8.9665	2.263467	0.049669	-3.92949
ILMN_1685052	4900612	DNAH1	-1.31146	6.725038	-2.26173	0.049811	-3.9306
ILMN_2257665	4390594	PARL	1.285133	9.094515	2.261055	0.049866	-3.93103
ILMN_1811909	2340739	LOC402221	1.255318	8.747358	2.260402	0.049919	-3.93144
ILMN_1750409	2940451	RAB9A	1.860887	9.249226	2.260287	0.049928	-3.93152

Table S3. PCR primers sequences

**Actin**	**5’ GGACTTCGAGCAAGAGATGG 3`**
	5’ AGCACTGTGTTGGCGTACAG 3`
**B-globine**	5’ GTGCATCTGACTCCTGAGGAG A 3`
	5’ AGCACACACACCAGCACATT 3`
**GAPDH**	F: 5’-AAGGTGGTGAAGCAGGCGT-3’
	R: 5’-GAGGAGTGGGTGTCGCTGTT-3’
**EBV-LMP1**	F: 5’-CCCCCTCTCCTCTTCCATAG-3’
	R: 5’-GCCAAAGATGAACAGCACAA-3’
**EBV-EBNA1**	F: 5’-GGACCCGGCCCACAACCTG-3’
	R: 5’-CTCCTGCCCTTCCTCACCCTCATC-3’
**TCF3**	F: 5’-CATGTGCATCCTCCTTCTCC-3’
	R: 5’-GAGTAGATCGAGGCCAGT GC-3’
**ID3**	F: 5’-CTGGACGACATGAACCACTG-3’
	R: 5’-AAGCTCCTTTTGTCGTTGGA-3’
